# Synthetic, Structural, and Anticancer Activity Evaluation Studies on Novel Pyrazolylnucleosides

**DOI:** 10.3390/molecules24213922

**Published:** 2019-10-30

**Authors:** Yogesh Yadav, Deepti Sharma, Kumar Kaushik, Vineet Kumar, Amitabh Jha, Ashok K. Prasad, Christophe Len, Sanjay V. Malhotra, Jesper Wengel, Virinder S. Parmar

**Affiliations:** 1 Bioorganic Laboratory, Department of Chemistry, University of Delhi, Delhi 110 007, India; yogeshyadav77@gmail.com (Y.Y.); iamvineet@gmail.com (V.K.); ashokenzyme@gmail.com (A.K.P.); 2 Medicinal Chemistry Laboratory, Department of Chemistry, Acadia University, Wolfville, NS B4P 2R6, Canada; amitabh.jha@acadiau.ca; 3 SUN Pharmaceuticals R&D, Gurgaon, Sarhaul, Sector-18, Haryana-122 015, India; 4 Sri Venkateswara College, Benito Juarez Road, Dhaula Kuan, University of Delhi, Delhi 110 021, India; 5 Department of Chemistry and Environmental Science, Medgar Evers College, The City University of New York, 1638 Bedford Avenue, Brooklyn, NY 11225, USA; kumar.kaushik.drdo@gmail.com; 6 Laboratory of Synthetic Chemistry, Leidos Biomedical Research Inc., Frederick National Laboratory for Cancer Research, Frederick, MD 2170, USA; smalhotra@stanford.edu; 7 Department of Radiation Oncology, Stanford University, 1050A Arastradero Road, A252, Palo Alto, CA 94304, USA; 8 Chimie ParisTech, PSL University, CNRS Institute of Chemistry for Life and Health Sciences—i-CLeHS, 11 rue Pierre et Marie Curie, F-75005 Paris, France; 9 Nucleic Acid Center, Department of Physics, Chemistry and Pharmacy, University of Southern Denmark, Campusvej 55, DK-5230 Odense, Denmark; jwe@sdu.dk

**Keywords:** pyrazolylnucleosides, modified nucleosides, NOESY study, anticancer, NCI-60

## Abstract

The synthesis of novel pyrazolylnucleosides **3a**–**e**, **4a**–**e**, **5a**–**e**, and **6a**–**e** are described. The structures of the regioisomers were elucidated by using extensive NMR studies. The pyrazolylnucleosides **5a**–**e** and **6a**–**e** were screened for anticancer activities on sixty human tumor cell lines. The compound **6e** showed good activity against 39 cancer cell lines. In particular, it showed significant inhibition against the lung cancer cell line Hop-92 (GI_50_ 9.3 µM) and breast cancer cell line HS 578T (GI_50_ 3.0 µM).

## 1. Introduction

**Figure 1:**
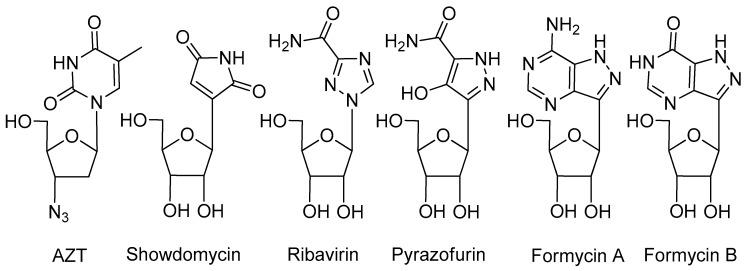
Selected nucleoside analogues with antiviral and/or antitumoral activities: 3’-azidothymidine (AZT or ZDV), showdomycin, ribavirin, pyrazofurin, formycin A and formycin B.

The chemistry of nucleosides has been extensively studied and several analogs have been found to exhibit potential as fungicidal, antitumor, and antiviral agents [1,2,3,4,5,6,7]. Modifications in both the heterocyclic bases and the sugar moieties have led to active and safer nucleoside analogues that have found applications as agents effective against human immunodeficiency virus (HIV), the causative agent of acquired immune deficiency syndrome (AIDS), and also against viral infections caused by the herpes simplex virus (HSV types 1 and 2), varicella zoster virus (VZV), hepatitis C virus (HCV), human cytomegalovirus (HCMV), and Epstein-Barr virus (EBV) [8,9]. Nucleoside and nucleotide modifications resulted in an increased interest in the regio- and stereoselective synthesis of nucleosides [10,11]. Moreover, modified nucleosides and nucleotides with a restricted conformation have been used to reach a particular conformation of a rotamer to study the affinity of a biomacromolecule for its natural ligand as well as the molecular recognition in an oligonucleotide chain (RNA/DNA) [12,13,14,15]. Similar studies of anti-sense and anti-gene oligonucleotides (ONs) as potential and selective inhibitors of gene expression [16,17,18,19] and their use as anti-tumor or anti-viral agents [20,21,22,23] have also influenced the developments in the field of nucleic acid-based drugs. Among the nucleoside analogues with significant biological activities, dideoxynucleoside-based compounds such as 2′,3**′**-dideoxycytidine (ddC) [24], 2**′**,3**′**-dideoxyinosine (ddI) [24], and 3**′**-azidothymidine (AZT) [25] are effective therapeutic agents for the treatment of AIDS, while ribavirin (virazole) [26,27] is an antiviral drug (Figure 1). Similarly, other dideoxynucleosides such as d4T (2**′**,3**′**-didehydro-3**′**-deoxythymidine, stavudine) [28,29] and AZddU (3**′**-azido-2**′**,3**′**-dideoxyuridine) [30] have gone through clinical studies. Different nucleosides isolated from nature such as oxazinomycin [31], pyrazofurin [32,33], showdomycin [34,35], formycin A, and formycin B [36,37] have shown antibiotic properties and have also been found to exhibit anticancer and/or antiviral activities (Figure 1). These examples and new developments in the chemistry and biology of these compounds and their analogs [38,39,40,41,42] have motivated us to work in this area and have led us to investigate the interesting chemical and biological properties of novel nucleoside-based compounds.

Despite the developments in nucleoside chemistry, the clinical use of nucleosides has some drawbacks due to their side-effects and primary or acquired drug resistance [43]. Therefore, the search for the design of new and effective nucleoside-based analogues continues to motivate studies in this field. Our efforts toward this goal have led to the synthesis of twenty novel pyrazolylnucleosides with new structures and potent antiviral and antitumoral behaviors. In this regard, two regioisomers for each pyrazole derivative have been produced and characterized using spectroscopic techniques such as ^1^H NMR, ^13^C NMR, NOESY, HMBC, IR, and mass spectroscopy. All compounds were evaluated against the National Cancer Institute (NCI)’s panel of 60 human tumor cell lines for their anticancer activities, and for their antiviral activities against representative viruses. 

## 2. Results and Discussion

### 2.1. Chemical Synthesis of the Nucleoside Analogues **5** and **6**

**Scheme 1:**
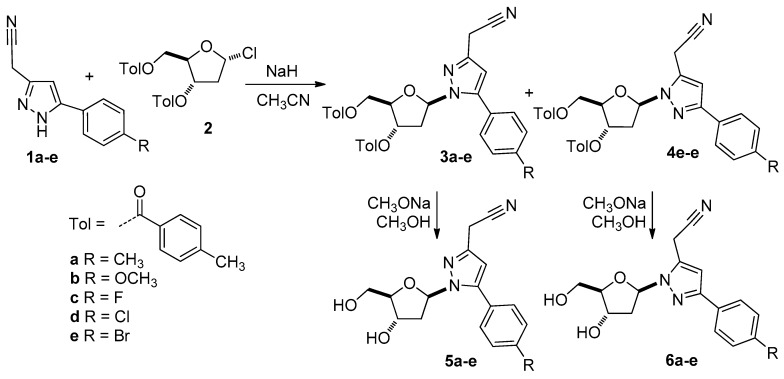
Synthesis of pyrazolylnucleoside analogues **5a**–**e** and **6a**–**e**.

In our approach, the synthesis of the target compounds **5a**–**e** and **6a**–**e** was achieved in two steps starting from the already reported 3-cyanomethyl-5-aryl-1*H*-pyrazoles **1a**–**e** [44] and the well-known 2-deoxy-3,5-di-*O*-*p*-toluoyl-*α*-d-ribofuranosyl chloride (**2**) [45] (Scheme 1). Treatment of pyrazoles **1a**–**e** with sodium hydride in acetonitrile and the subsequent addition of chlorosugar **2** gave two regioisomers of the modified pyrazolyl nucleosides **3a**–**e** (by coupling **2** with the *N*-1 nitrogen of the pyrazole derivatives) and **4a**–**e** (by coupling chlorosugar **2** with the *N*-2 nitrogen of the pyrazole derivatives) in 58–65% yields. The products **3a**–**e** and **4a**–**e** were de-toluoylated by using sodium methoxide in methanol, resulting in the formation of the corresponding modified nucleosides **5a**–**e** and **6a**–**e** in 70–80% yields, respectively. 

**Scheme 2:**
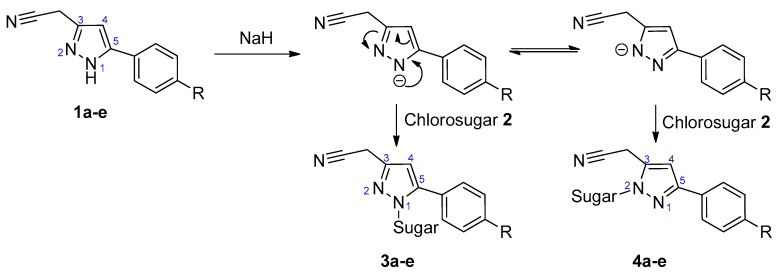
Mechanistic explanation for the regioisomeric alkylation of pyrazole derivatives **1a**–**e**. The IUPAC numbering scheme of **3a**–**e** is retained in **4a**–**e** (and in **5a**–**e** and **6a**–**e**) for easy comparison.

Pyrazoles with no substitution on either of the two N ring atoms can be alkylated to produce two regioisomers under strongly basic conditions. The anion generated produces the two resonance forms that react with chlorosugar **2** in the glycosidation step, leading to mixtures of two regioisomeric pyrazolyl nucleosides (i.e., **3a**–**e** and **4a**–**e**) (Scheme 2). 

### 2.2. Structural Identification of the Isomeric Pyrazolyl Nucleoside Analogues **3**–**6**

The structural identification of the isomeric disubstituted pyrazolyl nucleosides, based on their ^1^H NMR spectral data (See Supplementary Materials), has been reported in the literature [46,47,48]. It has been observed that the anomeric protons in the isomeric pyrazolyl nucleosides exhibit different proton chemical shifts. The anomeric proton adjacent to *N*-1 of the pyrazole compounds **3a**–**e** and **5a**–**e** would appear downfield when compared to the anomeric proton adjacent to *N*-2 of the pyrazole compounds **4a**–**e** and **6a**–**e** [46,47,48]. In addition to using ^1^H NMR chemical shifts, extensive NOE and 2D NMR experiments have been employed to confirm the structures of the isomeric pyrazolyl nucleosides. Using ^1^H and ^13^C NMR studies, we confirmed the positional assignments of the isomeric pyrazolyl nucleosides synthesized in the present work (Table 1, Table 2 and Table 3). The anomeric proton of the analogues **5a**–**e** generally appeared at 0.14–0.19 ppm upfield when compared to the corresponding proton in the ^1^H NMR spectra of its corresponding **6** series isomers. This is in agreement with observations for other 1,5- and 1,3-disubstituted pyrazole nucleosides [46,47,48]. We found that the effect was less pronounced in the **3** series of nucleosides as compared to the **4** series nucleosides where this difference was in the range of 0.04–0.14 ppm. The ^13^C chemical shift of the –CH_2_CN bearing pyrazole carbon atom can help distinguish between positional isomers. In the **3** and **5** series of nucleosides, this carbon signal was 4–11 ppm downfield relative to the corresponding signal in the **4** and **6** series nucleosides. In most cases, the aryl bearing pyrazole carbon in the **3** and **5** series was 4–7 ppm upfield relative to the corresponding carbon in the **4** and **6** series. However, in the case of **3d** versus **4d**, the difference was small and reversed, making the aryl bearing pyrazole carbon less reliable for use in positional assignments.

In order to confirm the above positional assignments, we performed 2D NMR experiments (NOESY and HMBC) on the two pairs of compounds (i.e., one pair consisting of the ditoluoyl protected nucleosides **3d** and **4d** and another pair consisting of the deprotected nucleosides **5d** and **6d**) (Table 4). The results of the HMBC experiments rely on the fact that the anomeric proton may see the aryl bearing pyrazole carbon in **3d** and **5d**, but not in **4d** and **6d**, while the NOESY results would explain the spatial proximity of the anomeric proton to either the protons in the –CH_2_CN group or to the *ortho* protons of the aryl group. Our results verified the positional properties at the pyrazole ring in the four compounds and are summarized in Table 4.

The isomers of the **3** series nucleosides generally had higher R_f_ values on the TLC than the corresponding isomer of the **4** series of nucleosides. The same was observed for the isomers of the **5** series relative to those in the **6** series of nucleosides. 

### 2.3. Anticancer Activity of the Isomeric Pyrazolyl Nucleoside Analogues **5a**–**e** and **6a**–**e**

The anticancer and toxicity of compounds **5a**–**e** and **6a**–**e** were evaluated by using the National Cancer Institute′s 60 human cancer cell lines. Compounds **5a**–**e**, where the sugar is attached to *N*-1 of the pyrazole ring, were found to be inactive against all the cell lines irrespective of the substituent on the aromatic ring. In the other series, compounds **6a**, **6b**, and **6c** with 4-methyl, 4-methoxy, and 4-fluoro substituents, respectively, at the aromatic ring were also inactive. However, compounds **6d** and **6e** with 4-chloro and 4-bromo substituents at the aromatic ring, respectively, showed inhibition against multiple anticancer cell lines. The GI_50_ (cytostatic parameter) and LC_50_ (toxicity parameter) values of **6d** and **6e** for the selected cell lines are given in Table 5. Compound **6d** showed inhibition in 19 cell lines, and was most active against the renal cancer cell line UO-31 and breast cancer cell line HS 578T with GI_50_ < 20 µM in both cases. The most active compound was **6e**, which showed moderate inhibition in 39 cell lines. It showed significant inhibition against lung cancer cell line Hop-92 with a GI_50_ of 9.3 µM and breast cancer cell line HS 578T with a GI_50_ of 3.0 µM. Most importantly, both compounds (**6d** and **6e**) did not show any toxicity, even at the highest concentration tested, as indicated by their high LC_50_ values. 

The pyrazolyl nucleosides **5a**–**e** and **6a**–**e** were also examined for their antiviral activities against a number of viruses such as Simplex virus type 1 (HSV-1) and type 2 (HSV-2), thymidine kinase-deficient (TK^-^) strains of HSV-1, Vaccinia virus, para-influenza-3 virus, Sindbis virus, Coxsackie virus, Punta toro virus, vesicular stomatitis virus (VSV), Coxsackie virus B4 (CV-B4), respiratory syncytial virus (RSV), feline corona virus, and feline herpes virus. However, none of these compounds showed any significant activity against any of these viruses.

## 3. Experimental

### 3.1. General Information

Reactions were conducted under an atmosphere of nitrogen in anhydrous solvents. Column chromatography was carried out by using silica gel (100–200 mesh). Melting points were determined in a concentrated H_2_SO_4_ acid bath and were uncorrected. Analytical TLCs were performed on pre-coated Merck silica gel 60F_254_ plates; the spots were detected either by using UV light or by charring with 4% alcoholic sulfuric acid. The IR spectra were recorded on a Perkin-Elmer 2000 FT-IR spectrophotometer (Waltham, MA, USA). The optical rotations were measured with a Bellingham-Stanley AD 220 polarimeter and the concentrations expressed as g/mL. The ^1^H-NMR and ^13^C-NMR spectra were recorded on a Bruker Avance 300 spectrometer (Billerica, MA, USA) at 300 and 75.5 MHz, respectively in CDCl_3_, DMSO-*d*_6_, or CD_3_CN. All 2D measurements were performed in acetone-*d*_6_ on a Bruker Avance 400 spectrometer (Billerica, MA, USA). Chemical shifts are relative to internal TMS. Assignments were based on COSY, NOESY, HMBC (by using Bruker’s microprogram inv4gslplrnd, which includes the low-pass J-filter to suppress one-bond correlations), HSQC, and JRES spectra. The chemical shift values were reported as δ ppm relative to TMS used as the internal standard and the coupling constants (*J*) were measured in Hz. The ESI-HRMS spectra of all compounds were recorded on a JEOL JMS-AX505W high-resolution mass spectrometer (Tokyo, Japan) in positive ion mode by using the matrix HEDS (*bis-*hydroxyethylsulfide) doped with sodium acetate. Acetonitrile was used after distillation over freshly ignited potassium carbonate.

### 3.2. Synthesis and Characterization

#### 3.2.1. Synthesis of 2′-Deoxy-1′-(3-cyanomethyl-5-aryl)pyrazolyl-3′,5′-di-*O*-toluoyl-β-d-ribofuranose (**3a**–**e**) and 2′-deoxy-1′-(3-aryl-5-cyanomethyl)pyrazolyl-3′,5′-di-*O*-toluoyl-β-d-ribofuranose (**4a**–**e**)

A solution of 3-cyanomethyl-5-arylpyrazole (**1a**–**e**, 15 mmol) in acetonitrile (190 mL) was added into a stirred mixture of sodium hydride (22.5 mmol) in acetonitrile (30 mL) under a nitrogen atmosphere at 30–35 °C, and continuously stirred for 30 min. The reaction mixture was cooled to 0 °C and 1-α-chloro-3,5-di-*O*-toluoyl-1,2-dideoxyribose (**2**, 15 mmol) was added and the contents stirred at 0 °C for 2.5 to 3 h. The progress of the reaction was monitored by using silica gel TLC. On completion, the reaction mixture was poured over ice-cold water and extracted with ethyl acetate (3 × 50 mL). The combined organic layer was washed with water (2 × 50 mL), dried over anhydrous sodium sulfate, the solvent was removed under reduced pressure, and the residue thus obtained was column chromatographed over silica gel with ethyl acetate (8–10%) in petroleum ether as the eluent to afford the pyrazolyl nucleosides **3a**–**e** and **4a**–**e** in 31 to 35% and 24 to 29% yields, respectively.

*2′-Deoxy-1′-(3-cyanomethyl-5-p-methylphenyl)pyrazolyl-3′,5′-di-O-toluoyl-β-d-ribofuranose* (**3a**): Obtained as a white semisolid in a 34% yield. ${\left[\alpha \right]}_{D}^{18}$ = −115.46° (*c* 0.25, CHCl_3_); R*_f_* = 0.53 (20% ethyl acetate in petroleum ether); IR (Nujol): 2923, 2256 (CN), 1721 (COO), 1611, 1452, 1269, 1177 cm^−1^; ^1^H NMR (300 MHz, CDCl_3_): δ 2.41 (9H, s, 3 × Ar-CH_3_), 2.45–2.51(1H, m, C-2**′**H_α_), 3.45–3.52 (1H, m, C-2**′**H_β_), 3.65 (2H, s, C*H_2_*CN), 4.55–4.67 (3H, m, C-4**′**H and C-5**′** H_α+β_), 5.86 (1H, brs, C-3**′**H), 6.13 (1H, t, *J* = 6.1 Hz, C-1**′**H), 6.33 (1H, s, C-4H), 7.21–7.24 (4H, brs, 4 × Ar-H), 7.28 (2H, d, *J* = 7.8 Hz, Ar-H), 7.42 (2H, d, *J* = 7.8 Hz, Ar-H), 7.90 (2H, d, *J* = 8.0 Hz, Ar-H), 8.00 (2H, d, *J* = 8.0, Ar-H); ^13^C NMR (75.5 MHz, CDCl_3_): δ 15.6, 19.3, 19.7, 19.7, 34.6, 62.4, 73.9, 80.6, 84.5, 103.8, 115.2, 124.4, 124.7, 125.2, 127.0, 127.1, 127.1, 127.5, 127.7, 127.9, 137.2, 139.9, 142.1, 144.6, 141.7, 164.0, 164.3; HRMS (ESI): Calculated for C_33_H_31_N_3_O_5_ [M + Na]^+^ 572.2156, found [M + Na]^+^ 572.2163.

*2′-Deoxy-1′-(3-cyanomethyl-5-p-methoxyphenyl)pyrazolyl-3′,5′-di-O-toluoyl-β-d-ribofuranose* (**3b**): Obtained as a semisolid in a 31% yield. ${\left[\alpha \right]}_{D}^{18}$ = −119.46° (*c* 0.25, CHCl_3_); R*_f_* = 0.31 (20% ethyl acetate in petroleum ether); IR (Nujol): 2926, 2256 (CN), 1724 (CO), 1610, 1505, 1254, 1178 cm^−1^; ^1^H NMR (300 MHz, CDCl_3_): δ 2.40 (6H, s, 2 × Ar-CH_3_), 2.46–2.50 (1H, m, C-2**′**H_α_), 3.48–3.53 (1H, m, C-2**′**H_β_), 3.64 (2H, s, C*H_2_*CN), 3.84 (3H, s, OCH_3_), 4.54–4.62 (3H, m, C- C-4**′**H & 5**′**H_α+β_), 5.87 (1H, brs, C-3**′**H), 6.10 (1H, brs, C-1**′**H), 6.30 (1H, s, C-4H), 6.99 (2H, d, *J =* 8.1, Ar-H), 7.21–7.23 (4H, m, Ar-H), 7.46 (2H, d, *J* = 8.0 Hz, Ar-H), 7.89 (2H, d, *J* = 7.6 Hz, Ar-H), 7.99 (2H, d, *J* = 7.6, Ar-H); ^13^C NMR (75.5 MHz, CDCl_3_): δ 19.7, 23.7, 23.8, 38.7, 57.5, 66.5, 78.0, 84.7, 88.6, 107.8, 116.4, 119.3, 123.8, 128.9, 129.4, 131.1, 131.2, 131.8, 132.0, 132.6, 144.0, 145.8, 146.2, 148.5, 162.4, 168.1, 168.4; HRMS (ESI): Calculated for C_33_H_31_N_3_O_6_ [M + Na]^+^ 588.2105, found [M + Na]^+^ 588.2127.

*2′-Deoxy-1′-(3-cyanomethyl-5-p-flurophenyl)pyrazolyl-3′,5′-di-O-toluoyl-β-d-ribofuranose* (**3c**): Obtained as a white semisolid in a 35% yield. ${\left[\alpha \right]}_{D}^{18}$ = −63.64° (*c* 0.25, CHCl_3_); R*_f_* = 0.54 (20% ethyl acetate in petroleum ether); IR (Nujol): 2925, 2256 (CN), 1723 (COO), 1610, 1506, 1453, 1177, 1104 cm^−1^; ^1^H NMR (300 MHz, CDCl_3_): δ 2.40 (6H, s, 2 × Ar-CH_3_), 2.48–2.52 (1H, m, C-2**′**H_α_), 3.50-3.54 (1H, m, C-2**′**H_β_), 3.64 (2H, s, C*H_2_*CN), 4.56–4.66 (3H, m, C-4**′**H & C-5**′**H), 5.87 (1H, brs, C-3**′**H), 6.05 (1H, t, *J* = 5.8 Hz, C-1**′**H), 6.33 (1H, s, C-4H), 7.13–7.24 (6H, m, Ar-H), 7.50–7.55 (2H, m, Ar-H), 7.90 (2H, d, *J* = 7.9 Hz, Ar-H), 7.98 (2H, d, *J =* 7.9 Hz, Ar-H); ^13^C NMR (75.5 MHz, CDCl_3_): δ 17.5, 21.5, 21.6, 36.4, 64.2, 75.7, 82.6, 86.3, 106.1, 115.9 (d, *J* = 22 Hz), 116.9, 125.4, 126.6, 127.1, 128.9, 129.0, 129.6, 129.8, 131.0 (d, *J* = 8 Hz), 141.9, 143.7, 144.1, 145.4, 163.1 (d, *J* = 247 Hz), 165.9, 166.1; HRMS (ESI): Calculated for C_32_H_28_N_3_O_5_F [M + Na]^+^ 576.1905, found [M + Na]^+^ 576.1916. 

*2′-Deoxy-1′-(3-cyanomethyl-5-p-chlorophenyl)pyrazolyl-3′,5′-di-O-toluoyl-β-d-ribofuranose* (**3d**): Obtained as a yellow solid in a 32% yield. ${\left[\alpha \right]}_{D}^{18}$ = −109.79° (*c* 0.25, CHCl_3_); Mp = 114–115 °C; R*_f_*= 0.52 (20% ethyl acetate in petroleum ether); IR (Nujol): 2924, 2257 (CN), 1728 (COO), 1610, 1494, 1177, 1108 cm^−1^; ^1^H NMR (300 MHz, CDCl_3_): δ 2.40 (6H, s, 2 × Ar-CH_3_), 2.46–2.52 (1H, m, C-2**′**H_α_), 3.50–3.57 (1H, m, C-2**′**H_β_), 3.63 (2H, s, C*H_2_*CN), 4.51–4.66 (3H, m, C-4**′**H & C-5**′**H), 5.86–5.88 (1H, m, C-3**′**H), 6.05 (1H, t, *J* = 6.1 Hz, C-1**′**H), 6.35 (1H, s, C-4H), 7.21–7.24 (4H, m, Ar-H), 7.43–7.50 (4H, m, Ar-H), 7.90 (2H, d, *J* = 7.9 Hz, Ar-H), 7.98 (2H, d, *J* = 7.9 Hz, Ar-H); ^13^C NMR (75.5 MHz, CDCl_3_): δ 17.4, 21.5, 21.6, 36.4, 64.1, 75.6, 82.7, 86.4, 106.1, 116.9, 126.4, 126.6, 127.6, 128.9, 129.0, 129.1, 129.6, 129.7, 130.3, 135.3, 141.9, 143.7, 144.1, 145.2, 165.9, 166.1; HRMS (ESI): Calculated for C_32_H_28_N_3_O_5_Cl [M + Na]^+^ 592.1610, found [M + Na]^+^ 592.1625.

*2′-Deoxy-1′-(3-cyanomethyl-5-p-bromophenyl)pyrazolyl-3′,5′-di-O-toluoyl-β-d-ribofuranose* (**3e**): Obtained as a white solid in a 35% yield. ${\left[\alpha \right]}_{D}^{18}$ = −96.35° (*c* 0.25, CHCl_3_); Mp = 118–110 °C; R*_f_* = 0.47 (20% ethyl acetate in petroleum ether); IR (Nujol): 2921, 2255 (CN), 1714 (COO), 1610, 1463, 1267, 1176, 1101 cm^−1^; ^1^H NMR (300 MHz, CDCl_3_): δ 2.41 (6H, s, 2 × Ar-CH_3_), 2.46–2.54 (1H, m, C-2**′**H_α_), 3.48–3.57 (1H, m, C-2**′**H_β_), 3.64 (2H, s, C*H_2_*CN), 4.51–4.66 (3H, m, C-4**′**H & C-5**′**H), 5.85–5.87 (1H, m, C-3**′**H), 6.05 (1H, t, *J* = 6.1 Hz, C-1**′**H), 6.35 (1H, s, C-4H), 7.22 (4H, d, *J* = 7.9 Hz, Ar-H), 7.42 (2H, d, *J* = 8.3 Hz, Ar-H), 7.60 (2H, d, *J* = 8.3 Hz, Ar-H), 7.90 (2H, d, *J* = 8.0 Hz, Ar-H), 7.98 (2H, d, *J* = 8.0 Hz, Ar-H); ^13^C NMR (75.5 MHz, CDCl_3_): δ 17.8, 21.9, 21.9, 36.8, 64.5, 76.0, 83.1, 86.8, 106.5, 117.3, 123.9, 127.0, 127.4, 128.5, 129.3, 129.4, 130.0, 130.1, 130.9, 132.4, 142.3, 144.0, 144.4, 145.6, 166.2, 166.5; HRMS (ESI): Calculated for C_32_H_28_N_3_O_5_Br [M + Na]^+^ 636.1105, found [M + Na]^+^ 636.1113.

*2′-Deoxy-1′-(3-p-methylphenyl-5-cyanomethyl)pyrazolyl-3′,5′-di-O-toluoyl-β-d-ribofuranose* (**4a**): Obtained as a white semisolid in a 26% yield. ${\left[\alpha \right]}_{D}^{18}$ = +25.10° (*c* 0.25, CHCl_3_); R*_f_* = 0.36 (20% ethyl acetate in petroleum ether); IR (Nujol): 2923, 2256 (CN), 1721 (COO), 1611, 1452, 1269, 1177 cm^−1^; ^1^H NMR (300 MHz, CDCl_3_): δ 2.31, 2.38 and 2.42 (9H, 3s, 3H each, 3 × Ar-CH_3_), 2.65-2.70 (1H, m, C-2**′**H_α_), 3.74–3.78 (1H, m, C-2**′**H_β_), 3.91 (2H, s, C*H_2_*CN), 4.36 (1H, q, *J* = 6.3 Hz, C-4**′**H), 4.54–4.60 (2H, m, C-5**′**H), 5.85-5.87 (1H, m, C-3**′**H), 6.17 (1H, t, *J* = 5.7 Hz, C-1**′**H), 6.59 (1H, s, C-4H), 7.03 (2H, d, *J* = 8.0 Hz, Ar-H), 7.18 (2H, d, *J* = 7.9 Hz, Ar-H), 7.26 (2H, d, *J* = 8.1 Hz, Ar-H), 7.63 (2H, d, *J* = 8.0 Hz, Ar-H), 7.83 (2H, d, *J* = 8.1 Hz, Ar-H), 7.95 (2H, d, *J* = 8.0 Hz, Ar-H); ^13^C NMR (75.5 MHz, CDCl_3_): δ 15.28, 21.2, 21.5, 21.6, 36.0, 63.6, 75.0, 83.2, 87.0, 105.3, 115.3, 125.6, 126.6, 126.7, 128.9, 129.0, 129.1, 129.2, 129.6, 129.7, 133.0, 137.9, 143.5, 144.2, 151.0, 165.9, 166.0; HRMS (ESI): Calculated for C_33_H_31_N_3_O_5_ [M + Na]^+^ 572.2156, found [M + Na]^+^ 572.2155.

*2′-Deoxy-1′-(3-p-methoxyphenyl-5-cyanomethyl)pyrazolyl-3′,5′-di-O-toluoyl-β-d-ribofuranose* (**4b**): Obtained as a white solid in a 24% yield. ${\left[\alpha \right]}_{D}^{18}$ = +42.05° (*c* 0.25, CHCl_3_); Mp = 88–90 °C; R*_f_*= 0.24 (20% ethyl acetate in petroleum ether); IR (Nujol): 2924, 2256 (CN), 1724 (COO), 1610, 1456, 1270, 1178 cm^−1^; ^1^H NMR (300 MHz, CDCl_3_): δ 2.32 and 2.42 (6H, 2s, 3H each, 2 × Ar-CH_3_), 2.65–2.69 (1H, m, C-2**′**H_α_), 3.73–3.77 (1H, m, C-2**′**H_β_), 3.83 (3H, s, Ar-OCH_3_), 3.90 (2H, s, C*H_2_*CN), 4.34–4.37 (1H, m, C-4**′**H), 4.56–4.59 (2H, m, C-5**′**H), 5.87 (1H, brs, C-3**′**H), 6.16 (1H, brs, C-1**′**H), 6.55 (1H, s, C-4H), 6.91 (2H, d, *J* = 7.6 Hz, Ar-H), 7.05 (2H, d, *J* = 7.3 Hz, Ar-H), 7.26 (2H, d, *J* = 6.9 Hz, Ar-H), 7.67 (2H, d, *J* = 7.7 Hz, Ar-H), 7.84 (2H, d, *J* = 7.3 Hz, Ar-H), 7.96 (2H, d, *J* = 7.4 Hz, Ar-H); ^13^C (75.5 MHz, CDCl_3_): δ 15.2, 21.5, 21.6, 36.0, 55.2, 63.6, 75.0, 83.1, 86.9, 105.0, 113.9, 115.4, 125.2, 126.6, 126.9, 128.9, 129.1, 129.5, 129.6, 129.7, 133.0, 143.5, 144.1, 150.8, 159.6, 165.8, 165.9; HRMS (ESI): Calculated for C_33_H_31_N_3_O_6_ [M + Na]^+^ 588.2105, found [M + Na]^+^ 588.2134.

*2′-Deoxy-1′-(3-p-flurophenyl-5-cyanomethyl)pyrazolyl-3′,5′-di-O-toluoyl-β-d-ribofuranose* (**4c**): Obtained as a white semisolid in a 25% yield. ${\left[\alpha \right]}_{D}^{18}$ = +14.07° (*c* 0.25, CHCl_3_); R*_f_* = 0.47 (20% ethyl acetate in petroleum ether); IR (Nujol): 2925, 2257 (CN), 1725 (COO), 1610,1522,1443,1272, 1178, 1102 cm^−1^; ^1^H NMR (300 MHz, CDCl_3_): δ 2.31, and 2.42 (6H, 2s, 3H each, 2 × Ar-CH_3_), 2.66–2.70 (1H, m, C-2**′**H_α_), 3.73–3.77 (1H, m, C-2**′**H_β_), 3.92 (2H, s, C*H_2_*CN), 4.33–4.35 (1H, m, C-4**′**H), 4.58–4.63 (2H, m, C-5**′**H), 5.87 (1H, brs, C-3**′**H), 6.17 (1H, t, *J* = 5.1 Hz, C-1**′**H), 6.56 (1H, s, C-4H), 7.02–7.08 (4H, m, Ar-H), 7.26 (2H, d, *J =* 7.9 Hz, Ar), 7.67–7.71 (2H, m, Ar-H), 7.83 (2H, d, *J* = 8.0 Hz, Ar-H), 7.96 (2H, d, *J* = 8.0 Hz, Ar-H); ^13^C NMR (75.5 MHz, CDCl_3_): δ 17.7, 23.9, 24.0, 38.5, 66.0, 77.3, 85.7, 89.5, 107.7, 117.8 (d, *J* = 22 Hz), 128.0, 129.0 (d, *J* = 7.5 Hz), 129.7, 129.8, 131.0, 131.4, 131.6, 132.0, 132.1, 135.8, 146.0, 146.7, 152.5, 165.4 (d, *J* = 250 Hz), 168.2, 168.4; HRMS (ESI): Calculated for C_32_H_28_N_3_O_5_F [M + Na]^+^ 576.1905, found [M + Na]^+^ 576.1915.

*2′-Deoxy-1′-(3-p-chlorophenyl-5-cyanomethyl)pyrazolyl-3′,5′-di-O-toluoyl-β-d-ribofuranose* (**4d**): Obtained as a yellow solid in a 24% yield. ${\left[\alpha \right]}_{D}^{18}$ = +34.06° (*c* 0.25, CHCl_3_); Mp = 109–110 °C; R*_f_* = 0.40 (20% ethyl acetate in petroleum ether); IR (nujol): 2924, 2257 (CN), 1716 (COO), 1459, 1377, 1176, cm^−1^; ^1^H NMR (300 MHz, CDCl_3_): δ 2.33 and 2.45 (6H, 2s, 3H each, 2 × Ar-CH_3_), 2.65–2.74 (1H, m, C-2**′**H_α_), 3.74–3.85 (1H, m, C-2**′**H_β_), 3.94 (2H, s, C*H_2_*CN), 4.33–4.38 (1H, m, C-4**′**H), 4.57–4.65 (2H, m, C-5**′**H), 5.88–5.90 (1H, m, C-3**′**H), 6.19 (1H, t, *J* = 5.8 Hz, C-1**′**H), 6.59 (1H, s, C-4H), 7.04 (2H, d, *J* = 7.9 Hz, Ar-H), 7.29 (2H, d, *J* = 8.0 Hz, Ar-H), 7.35 (2H, d, *J* = 8.4 Hz, Ar-H), 7.66 (2H, d, *J* = 8.4 Hz, Ar-H), 7.82 (2H, d, *J* = 8.1 Hz, Ar-H), 7.98 (2H, d, *J* = 8.0 Hz, Ar-H); ^13^C NMR (75.5 MHz, CDCl_3_): δ 15.7, 21.9, 22.0, 36.5, 63.9, 75.3, 83.8, 87.6, 105.8, 116.5, 127.0, 127.3, 129.1, 129.4, 129.6, 130.0, 130.1, 130.2, 131.3, 133.9, 134.3, 144.1, 144.7, 150.3, 166.2, 166.4; HRMS (ESI): Calculated for C_32_H_28_N_3_O_5_Cl [M + Na]^+^ 592.1610, found [M + Na]^+^ 592.1621.

*2′-Deoxy-1′-(3-p-bromophenyl-5-cyanomethyl-)pyrazolyl-3′,5′-di-O-toluoyl-β-d-ribofuranose* (**4e**): Obtained as a white solid in a 29% yield. ${\left[\alpha \right]}_{D}^{18}$ = +39.82° (*c* 0.25, CHCl_3_); Mp = 118–120 °C; R*_f_* = 0.25 (20% ethyl acetate in petroleum ether); IR (Nujol): 2923, 2255 (CN), 1716 (COO), 1611, 1464, 1270, 1177 cm^−1^; ^1^H NMR (300 MHz, CDCl_3_): δ 2.31 and 2.42 (6H, 2s, 3H each, 2 × Ar-CH_3_), 2.62–2.66 (1H, m, C-2**′**H_α_), 3.79–3.82 (1H, m, C-2**′**H_β_), 3.92 (2H, s, C*H_2_*CN), 4.32–4.36 (1H, m, C-4**′**H), 4.56–4.62 (2H, m, C-5**′**H), 5.86–5.88 (1H, m, C-3**′**H), 6.17 (1H, t, *J* = 5.7 Hz, C-1**′**H), 6.57 (1H, s, C-4H), 7.03 (2H, d, *J* = 7.9 Hz, Ar-H), 7.26 (2H, d, *J* = 7.9 Hz, Ar-H), 7.48 (2H, d, *J* = 8.3 Hz, Ar-H), 7.57 (2H, d, *J* = 8.4 Hz, Ar-H), 7.80 (2H, d, *J* = 8.0 Hz, Ar-H), 7.96 (2H, d, *J* = 8.0 Hz, Ar-H); ^13^C NMR (75.5 MHz, CDCl_3_): δ 15.2, 21.5, 21.6, 36.0, 63.4, 74.8, 83.3, 87.1, 105.3, 115.3, 122.0, 126.5, 126.6, 127.2, 128.9, 129.1, 129.5, 129.7, 131.3, 131.6, 133.5, 143.6, 144.2, 149.8, 166.0, 166.1; HRMS (ESI): Calculated for C_32_H_28_N_3_O_5_Br [M + Na]^+^ 636.1105, found [M + Na]^+^ 636.1116.

#### 3.2.2. Synthesis of 2′-Deoxy-1′-(3-cyanomethyl-5-aryl)pyrazolyl-β-D-ribofuranose (**5a**–**e**) and 2′-deoxy-1′-(3-aryl-5-cyanomethyl)pyrazolyl-β-d-ribofuranose (**6a**–**e**)

2′-Deoxy-1′-(3-cyanomethyl-5-aryl)pyrazolyl-3′,5′-di-*O*-toluoyl-*β*-d-ribofuranose (**3a**–**e**) or 2′-deoxy-1′-(3-aryl-5-cyanomethyl)pyrazolyl-3′,5′-di-*O*-toluoyl-*β*-d-ribofuranose (**4a**–**e**, 1.3 mmol) was suspended in dry methanol (18 mL), then a mixture of NaOMe and MeOH (10 mL, 1:3, v/v) was added to the resulting solution, and the contents stirred at room temperature for 3–4 h. The progress of the reaction was monitored by using silica gel TLC. On completion, NH_4_Cl was added to the reaction mixture to adjust the pH to 8. One-third of the solvent was removed under reduced pressure and the reaction mixture was poured in water and extracted with ethyl acetate (3 × 30 mL), the organic layer was dried over anhydrous sodium sulfate, the solvent was removed under reduced pressure, and the residue thus obtained was subjected to column chromatography over silica gel with methanol (2–2.5%) in chloroform as the eluent to afford 2′-deoxy-1′-(3-cyanomethyl-5-aryl)pyrazolyl-*β*-d-ribofuranose (**5a**–**e**) and 2′-deoxy-1′-(3-aryl-5-cyanomethyl)pyrazolyl-*β*-d-ribofuranose (**6a**–**e**).

*2′-Deoxy-1′-(3-cyanomethyl-5-p-methylphenyl)pyrazolyl-β-d-ribofuranose* (**5a**): Obtained as a white semisolid in a 63% yield. ${\left[\alpha \right]}_{D}^{30}$ = −87.56° (*c* 0.25, MeOH); R*_f_* = 0.3 (7% methanol in chloroform); IR (Nujol): 3391 (OH), 2936, 2258 (CN), 1613, 1506, 1456, 1253, 1180, 1055 cm^−1^; ^1^H NMR (300 MHz, CDCl_3_): δ 2.10–2.18 (1H, m, C-2**′**H_α_), 2.39 (3H, s, Ar-CH_3_), 2.87–2.91 (1H, m, C-2**′**H_β_), 3.53 (1H, brs, C-5**′**H_α_), 3.61 (1H, brs, C-5**′**H_β_), 3.84 (1H, brs, C-4**′**H), 3.90 (2H, s, C*H_2_*CN), 4.44 (1H, brs, C-3**′**H), 4.49 (1H, brs, C-3**′**OH), 5.13 (1H, brs, C-5**′**OH), 5.99 (1H, brs, C-1**′**H), 6.30 (1H, s, C-4H), 7.29 (2H, d, *J* = 7.3, Ar-H), 7.40 (2H, d, *J* = 7.7 Hz, Ar-H); ^13^C NMR (75.5 MHz, CDCl_3_): δ 17.3, 21.1, 39.9, 63.0, 71.6, 86.0, 88.3, 105.3, 117.7, 126.5, 128.9, 129.5, 138.8, 142.0, 145.9; HRMS (ESI): Calculated for C_17_H_19_N_3_O_3_ [M + Na]^+^ 336.1319, found [M + Na]^+^ 336.1288.

*2′-Deoxy-1′-(3-cyanomethyl-5-p-methoxyphenyl)pyrazolyl-β-d-ribofuranose* (**5b**): Obtained as a white semisolid in a 66% yield. ${\left[\alpha \right]}_{D}^{30}$ = −94.35° (*c* 0.25, MeOH); R*_f_* = 0.48 (7% methanol in chloroform); IR (Nujol): 3391 (OH), 2932, 2254 (CN), 1612, 1510, 1457, 1253, 1183, 1056 cm**^−^**^1^; ^1^H NMR (300 MHz, DMSO-*d*_6_): δ 2.17–2.21 (1H, m, C-2′H_α_), 2.89–2.93 (1H, m, C-2′H_β_), 3.57–3.61 (1H, m, C-5′H_α_), 3.65–3.70 (1H, m, C-5′H_β_), 3.83-3.85 (5H, m, Ar-OCH_3_ & C*H_2_*CN), 3.92-3.94 (1H, m, C-4′H), 4.53 (1H, brs, C-3′H), 4.64 (1H, t, *J* = 6.6, C-5′OH), 5.09 (1H, d, *J* = 4.1 Hz, C-3′OH), 6.02 (1H, t, *J* = 6.3 Hz, C-1′H), 6.26 (1H, s, C-4H), 7.00 (2H, d, *J* = 8.6 Hz, Ar-H), 7.42 (2H, d, *J* = 8.6 Hz, Ar-H); ^13^C NMR (75.5 MHz, DMSO-*d*_6_): δ 17.3, 39.9, 55.3, 63.1, 71.8, 86.0, 88.4, 105.1, 114.3, 117.4, 121.5, 130.3, 141.8, 145.8, 160.1; HRMS (ESI): Calculated C_17_H_19_N_3_O_4_ [M + Na]^+^ 352.1268, found [M + Na]^+^ 352.1248.

*2′-Deoxy-1′-(3-cyanomethyl-5-p-fluorophenyl)pyrazolyl-β-d-ribofuranose* (**5c**): Obtained as a semisolid in a 63% yield. ${\left[\alpha \right]}_{D}^{30}$ = −153.52° (*c* 0.25, MeOH); R*_f_* = 0.41 (7% methanol in chloroform); IR (Nujol): 3393 (OH), 2932, 2256 (CN), 1611, 1508, 1451, 1255, 1182, 1051 cm^−1^; ^1^H NMR (300 MHz, DMSO-*d*_6_): δ 2.18–2.22 (1H, m, C-2**′**H_α_), 2.91–2.95 (1H, m, C-2**′**H_β_), 3.61 (1H, brs, C-5**′**H_α_), 3.65 (1H, brs, C-5**′**H_β_), 3.87 (2H, s, C*H_2_*CN), 3.93 (1H, brs, C-4**′**H), 4.53 (2H, brs, C-3**′**H and OH), 5.08 (1H, brs, C-5**′**OH), 5.97 (1H, brs, C-1**′**H), 6.32 (1H, s, C-4H), 7.22 (2H, brs, Ar-H), 7.53 (2H, brs, Ar-H); ^13^C NMR (75.5 MHz, DMSO-*d*_6_): δ 17.3, 39.9, 63.0, 71.7, 86.1, 88.4, 105.7, 115.9 (d, *J* = 22 Hz), 117.3, 125.6, 131.1 (d, *J* = 8 Hz), 141.9, 144.9, 162.8 (d, *J* = 246 Hz); HRMS (ESI): Calculated for C_16_H_16_N_3_O_3_F [M + Na]^+^ 340.1068, found [M + Na]^+^ 340.1058.

*2′-Deoxy-1′-(3-cyanomethyl-5-p-chlorophenyl)pyrazolyl-β-d-ribofuranose* (**5d**): Obtained as a semisolid in a 63% yield. ${\left[\alpha \right]}_{D}^{30}$ = −124.74° (*c* 0.25, MeOH); R*_f_* = 0.46 (7% methanol in chloroform); IR (Nujol): 3393 (OH), 2932, 2256 (CN), 1611, 1498, 1451, 1254, 1181, 1048 cm^−1^; ^1^H NMR (300 MHz, DMSO-*d_6_*): δ 2.22–2.24 (1H, m, C-2**′**H_α_), 2.92–2.96 (1H, m, C-2**′**H_β_), 3.59–3.61 (1H, m, C-5**′**H_α_), 3.68–3.72 (1H, m, C-5**′**H_β_), 3.85 (2H, brs, C*H_2_*CN), 3.97 (1H, brs, C-4**′**H), 4.57 (2H, brs, C-3**′**H and OH), 5.09 (1H, brs, C-5**′**OH), 6.00 (1H, brs, C-1**′**H), 6.34 (1H, s, C-4H), 7.24 (2H, d, *J* = 7.5 Hz, Ar-H), 7.87 (2H, d, *J* = 8.5 Hz, Ar-H); ^13^C (75.5 MHz, DMSO-*d*_6_): δ 17.3, 39.9, 63.1, 71.8, 86.2, 88.5, 105.7, 117.8, 127.8, 129.3, 130.4, 134.7, 141.9, 144.7; HRMS (ESI): Calculated for C_16_H_16_N_3_O_3_Cl [M + Na]^+^ 356.0772, found [M + Na]^+^ 356.0770.

*2′-Deoxy-1′-(3-cyanomethyl-5-p-bromophenyl)pyrazolyl-β-d-ribofuranose* (**5e**): Obtained as a semisolid in a 64% yield. ${\left[\alpha \right]}_{D}^{30}$ = −105.15° (*c* 0.25, MeOH); R*_f_* = 0.53 (7% methanol in chloroform); IR (Nujol): 3387 (OH), 2935, 2249 (CN), 1612, 1504, 1454, 1248, 1179, 1048 cm^−1^; ^1^H NMR (300 MHz, DMSO-*d*_6_): δ 2.20–2.24 (1H, m, C-2**′**H_α_), 2.90–2.96 (1H, m, C-2**′**H_β_), 3.59–3.61 (1H, m, C-5**′**H_α_), 3.68–3.72 (1H, m, C-5**′**H_β_), 3.85 (2H, brs, C*H_2_*CN), 3.97 (1H, brs, C-4**′**H), 4.56 (2H, brs, C-3**′**H & OH ), 5.07 (1H, brs, C-5**′**OH), 6.00 (1H, t, *J* = 6.06 Hz, C-1**′**H), 6.34 (1H, s, C-4H), 7.42 (2H, d, *J* = 8.1 Hz, Ar-H), 7.61 (2H, d, *J* = 8.0 Hz, Ar-H); ^13^C (75.5 MHz, DMSO-*d*_6_): δ 16.5, 39.0, 62.2, 71.0, 85.3, 87.6, 104.8, 116.3, 122.2, 128.2, 129.8, 131.0, 141.1, 143.9; HRMS (ESI): Calculated for C_16_H_16_N_3_O_3_Br [M + Na]^+^ 400.0267, found [M + Na]^+^ 400.0232.

*2′-Deoxy-1′-(5-cyanomethyl-3-p-methylphenyl)pyrazolyl-β-d-ribofuranose* (**6a**): Obtained as a white semisolid in a 61% yield. ${\left[\alpha \right]}_{D}^{30}$ = +34.78° (*c* 0.25, MeOH); R*_f_* = 0.3 (7% methanol in chloroform); IR (Nujol): 3392 (OH), 2934, 2258 (CN), 1611, 1504, 1452, 1254, 1179, 1051 cm**^−^**^1^; ^1^H NMR (300 MHz, CDCl_3_): δ 2.23–2.27 (1H, m, C-2′H_α_), 2.31 (3H, s, Ph-CH_3_), 2.77–2.83 (1H, m, C-2′H_β_), 3.36–3.40 (1H, m, C-5′H_α_), 3.49–3.51 (1H, m, C5′H_β_), 3.82–3.86 (1H, m, C-4′H), 4.32 (2H, s, C*H_2_*CN), 4.46 (1H, brs, C-3′H), 4.86 (1H, t, *J* = 5.4 Hz, C-5′OH), 5.29 (1H, d, *J* = 4.0 Hz, C-3′OH), 6.13 (1H, t, *J* = 5.9 Hz, C-1′H), 6.72 (1H, s, C-4H), 7.22 and 7.69 (4H, 2d, 2H each, *J* = 7.8 Hz, & 7.9 Hz, ArH); ^13^C NMR (75.5 MHz, DMSO-*d*_6_): δ 14.6, 21.1, 39.8, 62.6, 71.4, 86.5, 88.4, 104.0, 117.5, 125.5, 129.6, 130.1, 135.0, 137.7, 150.2; HRMS (ESI): Calculated for C_17_H_19_N_3_O_3_ [M + Na]^+^ 336.1319, found [M + Na]^+^ 336.1290.

*2′-Deoxy-1′-(5-cyanomethyl-3-p-methoxyphenyl)pyrazolyl-β-d-ribofuranose* (**6b**): Obtained as a semisolid in a 62% yield. ${\left[\alpha \right]}_{D}^{30}$ = +56.37^o^ (*c* 0.25, MeOH); R*_f_* = 0.39 (7% methanol in chloroform); IR (Nujol): 3385 (OH), 2937, 2258 (CN), 1610, 1501, 1448, 1251, 1183, 1053 cm**^−^**^1^; ^1^H NMR (300 MHz, DMSO-*d*_6_): δ 2.40–2.42 (1H, m, C-2′H_α_), 2.94–2.98 (1H, m, C-2′H_β_), 3.60–3.64 (1H, m, C-5′H_α_), 3.72–3.76 (1H, m, C-5′H_β_), 3.81 (3H, s, Ph-OCH_3_), 3.87 (1H, brs, C-4′H), 4.08 (2H, s, C*H_2_*CN), 4.61 (1H, brs, C-3′H), 5.01–5.05 (2H, m, C-5′OH & C-3′OH), 6.19 (1H, t, *J* = 6.0 Hz, C-1′H), 6.56 (1H, s, C-4H), 6.91 and 7.66 (4H, 2d, 2H each, *J* = 8.4 Hz and 8.4 Hz, Ar-H); ^13^C NMR (75.5 MHz, DMSO-*d*_6_): δ 14.9, 39.9, 55.1, 63.2, 71.9, 86.9, 88.9, 103.6, 114.0, 116.1, 126.7, 129.3, 133.7, 150.7, 159.6; HRMS (ESI): Calculated for C_17_H_19_N_3_O_4_ [M + Na]^+^ 352.1268, found [M + Na]^+^ 352.1237.

*2′-Deoxy-1′-(5-cyanomethyl-3-p-fluorophenyl)pyrazolyl-β-d-ribofuranose* (**6c**): Obtained as a semisolid in a 59% yield. ${\left[\alpha \right]}_{D}^{30}$ = +47.97° (*c* 0.25, MeOH); R*_f_* = 0.36 (7% methanol in chloroform); IR (Nujol): 3391 (OH), 2928, 2249 (CN), 1610, 1503, 1448, 1252, 1179, 1055 cm**^−^**^1^; ^1^H NMR (300 MHz, CDCl_3_): δ 2.24–2.28 (1H, m, C-2′H_α_), 2.85–2.89 (1H, m, C-2′H_β_), 3.39–3.41 (1H, m, C-5′H_α_), 3.48–3.54 (1H, m, C-5′ H_β_), 3.82–3.87 (1H, m, C-4′H), 4.33 (2H, s, C*H_2_*CN), 4.46 (1H, brs, C-3′H), 4.81 (1H, t, *J* = 5.4 Hz, C-5′OH), 5.27 (1H, d, *J* = 4.0 Hz, C-3′OH), 6.16 (1H, t, *J* = 6.0 Hz, C-1′H), 6.76 (1H, s, C-4H), 7.24 (2H, t, *J* = 7.6 Hz), 7.83 (2H, q, *J* = 5.3 Hz, Ar-H); ^13^C NMR (75.5 MHz, DMSO-*d_6_*): δ 15.2, 40.3, 63.1, 71.9, 87.0, 89.0, 104.7, 116.5 (d, *J* = 22 Hz), 117.9, 128.1 (d, *J* = 8 Hz), 130.0, 135.8, 149.8, 162.9 (d, *J* = 243 Hz); HRMS (ESI): Calculated for C_16_H_16_N_3_O_3_F [M + Na]^+^ 340.1068, found [M + Na]^+^ 340.1038.

*2′-Deoxy-1′-(3-p-chlorophenyl-5-cyanomethyl-)pyrazolyl-β-d-ribofuranose* (**6d**): Obtained as a semisolid in a 61% yield. ${\left[\alpha \right]}_{D}^{30}$ = +37.58° (*c* 0.25, MeOH); R*_f_* = 0.36 (7% methanol in chloroform); IR (Nujol): 3387 (OH), 2935, 2249 (CN), 1612, 1504, 1454, 1248, 1179, 1048 cm**^−^**^1^; ^1^H NMR (300 MHz, CD_3_CN): δ 2.36–2.43 (1H, m, C-2′H_α_), 2.88–2.96 (1H, m, C-2′H_β_), 3.43 (1H, brs, C-4′H), 3.56–3.63 (1H, m, C-5′H_α_), 3.69–3.73 (1H, m, C-5′ H_β_), 4.05–4.11 (4H, m, C*H_2_*CN, C-3′H & C-5′OH), 4.62 (1H, brs, C-3′OH), 6.17 (1H, t, *J* = 6.1 Hz, C-1′H), 6.72 (1H, s, C-4H), 7.44 (2H, d, *J* = 8.5 Hz, Ar-H), 7.77 (2H, d, *J* = 8.5 Hz, Ar-H); ^13^C NMR (75.5 MHz, CD_3_CN): δ 14.4, 39.9, 62.6, 71.7, 86.6, 88.6, 103.7, 116.9, 126.6, 128.5, 130.9, 133.1, 134.6, 149.6; HRMS (ESI): Calculated for C_16_H_16_N_3_O_3_Cl [M + Na]^+^ 356.0772, found [M + Na]^+^ 356.0757.

*2′-Deoxy-1′-(3-p-bromophenyl-5-cyanomethyl)pyrazolyl-β-d-ribofuranose* (**6e**): Obtained as a white solid in a 63% yield. ${\left[\alpha \right]}_{D}^{30}$ = +50.37° (*c* 0.25, MeOH); Mp = 74–75 °C; R*_f_* = 0.39 (7% methanol in chloroform); IR (Nujol): 3385 (OH), 2937, 2258 (CN), 1610, 1501, 1448, 1251, 1183, 1053 cm**^−^**^1^; ^1^H NMR (300 MHz, DMSO-*d*_6_): δ 2.34–2.39 (1H, m, C-2′H_α_), 2.92–2.98 (1H, m, C-2′H_β_), 3.47 (1H, brs, C-4′H), 3.58 (1H, brs, C-5′H_α_), 3.95 (1H, brs, C-5′ H_β_), 4.24 (2H, s, C*H_2_*CN), 4.51 (1H, brs, 3′H), 4.77 (1H, brs, C-3′OH), 5.20 (1H, brs, C-5′OH), 6.18 (1H, s, C-1′H), 6.71 (1H, s, C-4H), 7.55 (2H, brs, Ar-H), 7.69 (2H, brs, Ar-H); ^13^C NMR (75.5 MHz, DMSO-*d*_6_): δ 14.9, 39.7, 62.7, 71.4, 86.9, 88.6, 104.3, 116.8, 121.5, 127.3, 129.2, 131.7, 134.9, 149.2; HRMS (ESI): Calculated for C_16_H_16_N_3_O_3_Br [M + Na]^+^ 400.0267, found [M + Na]^+^ 400.0234.

### 3.3. NCI-60 Human Tumor Cell Line Screen

Details of the methodology are described at http://dtp.nci.nih.gov/branches/btb/ivclsp.html. Briefly, the panel was organized into nine subpanels representing a diverse histology: leukemia, melanoma, and cancers of the lung, colon, kidney, ovary, breast, prostate, and central nervous system. The cells were grown in supplemented RPM1 1640 medium for 24 h. For the five dose study, the test compounds were dissolved in DMSO and incubated with cells at five concentrations with 10-fold dilutions, the highest being 10^−4^ M and the others being 10^−5^, 10^−6^, 10^−7^, and 10^−8^ M. The assay was terminated by the addition of cold trichloroacetic acid, and the cells were fixed and stained with sulforhodamine B. The bound stain was solubilized, and the absorbance was read on an automated plate reader. The cytostatic parameter, which determines the 50% growth inhibition (GI_50_) of the tumor cells, was calculated from time zero, control growth, and the absorbance at the five concentration levels. The cytotoxic parameter, lethal concentration (LC_50_ is the concentration of a drug resulting in a 50% reduction in the measured protein at the end of the drug treatment when compared to that at the beginning), indicating a net loss of cells following the treatment, was calculated as the average of two independent experiments.

## 4. Conclusions

We successfully achieved the synthesis of twenty novel pyrazolyl nucleosides **3a**–**e**, **4a**–**e**, **5a**–**e**, and **6a**–**e**, which have been characterized in detail by using various NMR spectroscopic techniques such as ^1^H NMR, ^13^C NMR, NOESY, HMBC, etc. The pyrazolyl nucleosides **5a**–**e** and **6a**–**e** were screened for anticancer activities on 60 human tumor cell lines, and we identified one compound **6e**, which showed good activity against 39 cancer cell lines and showed significant inhibition against lung cancer cell line Hop-92 (GI_50_ 9.3 µM) and breast cancer cell line HS 578T (GI_50_ 3.0 µM). Our studies have demonstrated the potential of newly synthesized pyrazolyl-nucleosides that will be pursued further. 

## Figures and Tables

**Table 1:** Chemical shift values of the anomeric protons in the ^1^H NMR spectra and chemical shift values of the Ar- and CH_2_CN bearing carbons of the pyrazole ring in the ^13^C NMR spectra of the isomeric pyrazolyl nucleosides **3a**–**e**, **4a**–**e**, **5a**–**e**, and **6a**–**e** in CDCl_3_ on a Bruker Avance 300 spectrometer.

Compound	C-1′ H Shift in the ^1^H NMR Spectrum (ppm)		Ar- Bearing C Shifts in the ^13^C NMR Spectrum (ppm)		–CH_2_CN Bearing C Shifts in the ^13^C NMR Spectrum (ppm)	
	Series 3	Series 5	Series 3	Series 5	Series 3	Series 5
**a**	6.13	5.99	144.6	145.9	141.7	142.0
**b**	6.10	6.02	146.2	145.8	144.0	141.8
**c**	6.05	5.97	145.4	144.9	141.9	141.9
**d**	6.05	6.00	145.2	144.7	143.7	141.9
**e**	6.05	6.00	145.6	143.9	142.3	141.1
	**Series 4**	**Series 6**	**Series 4**	**Series 6**	**Series 4**	**Series 6**
**a**	6.17	6.13	151.0	150.2	137.9	137.7
**b**	6.16	6.19	150.8	150.7	133.0	133.7
**c**	6.17	6.16	152.5	149.8	135.8	135.8
**d**	6.19	6.17	144.1	149.6	134.3	134.6
**e**	6.17	6.18	149.8	149.2	133.5	134.9

**Table 2:** Chemical shift and coupling constants from the ^1^H-NMR of compounds **3d**, **4d**, **5d,** and **6d** in acetone-*d*_6_.^a^ Please refer to structures in Table 4 for skeleton numbering.

Proton	Chemical Shifts (δ)				Coupling Constants (Hz)			
	3d	4d	5d	6d	3d	4d	5d	6d
H1′	6.29	6.48	6.09	6.29	dd 5.7, 6.5	dd 6.5, 5.2	t* 6.4	t* 6.2
H2′α	2.72	2.79	2.31	2.42	ddd 14.0, 6.7, 4.1	ddd 14.0, 4.5, 6.6	ddd 13.5, 3.7, 6.8	ddd 13.5, 3.7, 6.7
H2′β	3.54	3.63	3.01	3.00	ddd 14.0, 6.8, 5.6	ddd 14.0, 5.2, 6.7	dt* 13.5, 6.0	dt* 13.5, 5.9
H3′	5.93	5.97	4.66	4.70	m	m	m	m
H4′	4.62	4.65	4.02	4.07	m	m	m	m
H5′α	4.54	4.45	3.61	3.59	m	dd 13.2, 6.4	ddd 11.8, 4.8, 7.7	ddd 11.9, 4.1, 8.1
H5′β	4.63	4.63	3.70	3.71	m	m	dt* 11.9, 4.7	dt* 11.9,4.1
H4	6.50	6.82	6.46	6.83	brs, 1H	t* 0.8	t* 0.5	t 0.8
H6	3.90	4.29	3.96	4.31	brs, 2H	19.0, 0.8	d 0.5	18.9, 0.8
H2″	7.67	7.87	7.60	7.87				
H3″			7.57	7.46				

^a^ The chemical shift values presented in Table 2 were all measured in acetone-*d*_6_ (on an Bruker Avance 400 instrument) and may therefore differ from those in Table 1, which were measured in CDCl_3_ (on Bruker Avance 300 instrument). * Due to the spatial interactions between different protons as explicitly shown in Table 4, these protons exhibited multiplicities as shown here when the spectra were recorded in acetone-*d*_6_ on a Bruker Avance 400 instrument as against those recorded in CDCl_3_, CD_3_CN or DMSO-*d*_6_ as given in the Experimental section for the corresponding protons where they appeared as broad singlets or ill resolved multiplets when their spectra were recorded on a Bruker Avance 300 instrument.

**Table 3:** Chemical shift values from the ^13^C-NMR of compounds **3d**, **4d**, **5d,** and **6d** in acetone-*d*_6_.^a^ Please refer to the structures in Table 4 for skeleton numbering.

Chemical Shifts (δ) (Carbon Position)			
3d	5d	4d	6d
87.6 (C1′)	87.4 (C1′)	87.6 (C1′)	88.0 (C1′)
37.1 (C2′)	40.5 (C2′)	37.0 (C2′)	41.1 (C2′)
76.6 (C3′)	73.2 (C3′)	76.1 (C3′)	73.0 (C3′)
83.5 (C4′)	89.8 (C4′)	83.7 (C4′)	90.0 (C4′)
65.1 (C5′)	64.2 (C5′)	64.7 (C5′)	63.9 (C5′)
143.8 (C3)	143.6 (C3)	136.3 (C3)	136.1 (C3)
107.2 (C4)	106.7 (C4)	105.4 (C4)	104.9 (C4)
146.0 (C5)	145.8 (C5)	150.6 (C5)	150.5 (C5)
17.6 (C6)	17.6 (C6)	15.3 (C6)	15.2 (C6)
118.1 (CN)	118.0 (CN)	117.0 (CN)	117.1 (CN)
166.4 (C6′′′)		166.5 (C6′′′)	
166.5 (C6′′′′)		166.5 (C6′′′′)	
*	129.3 (C1′′)	#	134.2 (C1′′)
*	131.6 (C2′′)	#	127.9 (C2′′)
*	129.9 (C3′′)	#	129.7 (C3′′)
*	135.6 (C4′′)	#	132.6 (C4′′)

^a^ The chemical shift values presented in Table 3 were all measured in acetone-d_6_ (on an Bruker Avance 400 instrument) and may therefore differ from those in Table 1, which were measured in CDCl_3_ (on Bruker Avance 300 instrument). * Benzene ring *Carbons*: Quaternary C: 145.0, 144.7, 135.6, 129.3, 128.3, 128.1; CHs: 131.6, 130.5, 130.5, 130.0, 130.0, 129.9. *# Benzene ring* *Carbons*: Quaternary C: 145.0, 144.5, 134.1, 132.7, 128.1; CHs: 130.5, 130.4, 130.1, 129.9, 129.5.

**Table 4:** Results from the NOESY and HMBC spectra of the pairs **3d**–**4d** and **5d**–**6d** *.

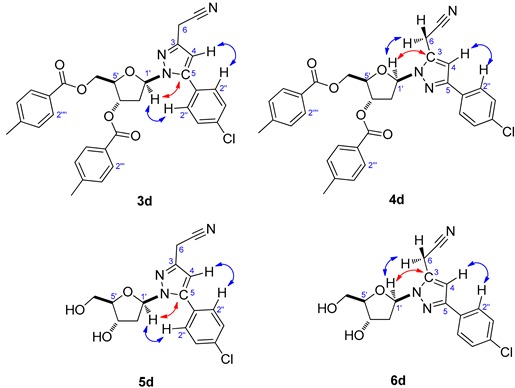 Blue and red arrows indicate the NOESY and HMBC cross peaks, respectively.				
	**3d**	**4d**	**5d**	**6d**
NOESY H1′-H6	o	x	o	x
NOESY H1′-H2′′	x	o	x	o
NOESY H4-H2′′	x	x	x	x
HMBC H1′-C5	x	o	x	o
HMBC H1′-C3	o	x	o	x

* The presence of a cross peak is indicated by (x). When it contributes to the positional verification, it is underlined (x). See Table 1, Table 2 and Table 3 for positions of the relevant protons and carbons in the NMR spectra of the compounds **3d**, **4d**, **5d** and **6d**.

**Table 5:** Antitumor activity (GI_50_/µM) ^a^ and toxicity (LC_50_/µM) ^b^ data of **6d** and **6e** with the anticancer drug dasatinib as a positive control.

Panels/Cell Lines	6d		6e		Dasatinib	
	GI_50_	LC_50_	GI_50_	LC_50_	GI_50_	LC_50_
**Leukemia**						
CCRF-CEM	>67.5	>67.5	46.3	>57.5	5.3	>100
K-562	>67.5	>67.5	40.0	>57.5	0.01	>100
MOLT-4	>67.5	>67.5	30.9	>57.5	4.1	>100
RPMI-8226	>67.5	>67.5	34.0	>57.5	4.8	99.5
SR	>67.5	>67.5	25.5	>57.5	3.1	89.3
**Non-Small Cell Lung Cancer**						
A549/ATCC	44.8	>67.5	29.3	>57.5	0.05	75.5
HOP-92	>67.5	>67.5	9.3	>57.5	0.01	>100
NCI-H322M	29.8	>67.5	30.2	>57.5	0.04	27.0
NCI-H522	>67.5	>67.5	25.7	>57.5	0.06	55.1
**Colon Cancer**						
HCT-116	>67.5	>67.5	47.7	>57.5	11.8	69.8
HCT-15	>67.5	>67.5	45.1	>57.5	0.6	71.1
**CNS Cancer**						
SF-268	33.5	>67.5	20.5	>57.5	0.07	75.5
SF-295	>67.5	>67.5	24.8	>57.5	1.1	46.8
SNB-19	24.6	>67.5	18.5	>57.5	11.9	76.4
SNB-75	53.5	>67.5	10.5	>57.5	0.01	46.3
U251	>67.5	>67.5	36.4	>57.5	3.2	52.4
**Melanoma**						
LOX IMVI	>67.5	>67.5	49.7	>57.5	0.01	2.8
M14	>67.5	>67.5	57.1	>57.5	3.1	51.5
MDA-MB-435	>67.5	>67.5	53.0	>57.5	4.1	65.5
SK-MEL-2	>67.5	>67.5	39.6	>57.5	1.1	71.8
SK-MEL-5	60.9	>67.5	11.8	>57.5	4.5	44.3
UACC-62	37.8	>67.5	10.5	>57.5	2.8	47.0
**Ovarian Cancer**						
IGROV1	32.6	>67.5	21.5	>57.5	0.01	80.0
OVCAR-3	>67.5	>67.5	39.1	>57.5	0.2	74.6
OVCAR-5	>67.5	>67.5	44.4	>57.5	0.02	86.3
**Renal Cancer**						
A498	22.6	>67.5	18.8	>57.5	0.03	16.0
ACHN	>67.5	>67.5	45.1	>57.5	0.01	520
CAK-1	22.9	>67.5	18.8	>57.5	0.01	5.1
RXF 393	38.0	>67.5	34.6	>57.5	0.03	10.4
SN12C	48.4	>67.5	24.3	>57.5	0.05	44.4
UO-31	19.4	>67.5	16.6	>57.5	0.01	82.0
**Prostate Cancer**						
PC-3	37.4	>67.5	20.0	>57.5	0.2	92.5
DU-145	>67.5	>67.5	45.1	>57.5	0.1	4.9
**Breast Cancer**						
MCF7	48.0	>67.5	32.8	>57.5	12.6	71.4
MDA-MB-231ATCC	>67.5	>67.5	55.8	>57.5	0.01	38.0
HS 578T	16.6	>67.5	3.0	>57.5	0.01	>100
BT-549	49.9	>67.5	30.0	>57.5	5.9	49.8
T-47D	26.6	>67.5	19.4	>57.5	0.2	91.4
MDA-MB-468	31.6	>67.5	13.8	>57.5	0.09	8.4

^a^ GI_50_: 50% Growth inhibition, concentration of drug resulting in a 50% reduction in net protein increase when compared with the control cells. ^b^ LC_50_: Lethal concentration, concentration of drug lethal to 50% of cells.

## Supplementary Materials

The following are available online at https://www.mdpi.com/1420-3049/24/21/3922/s1, Scanned copies of the NMR and IR spectra of selected compounds, as appropriate are given in the “Supplementary Information”.
