# Supplementary material for: Synthetic, Structural, and Anticancer Activity Evaluation Studies on Novel Pyrazolylnucleosides
Source: Molecules. 2019 Oct 30;24(21):3922. doi: 10.3390/molecules24213922 (PMC6864788; doi:10.3390/molecules24213922)
Supplement: Supplementary file 1 [file molecules-24-03922-s001.pdf]

SUPPORTING INFORMATION OF THE SYNTHETIC, STRUCTURAL AND ANTICANCER ACTIVITY EVALUATION STUDIES ON NOVEL PYRAZOLYLNUCLEOSIDES

EXPERIMENTAL

Reactions were conducted under an atmosphere of nitrogen in anhydrous solvents. Column chromatography was carried out using silica gel (100-200 mesh). Melting points were determined using concentrated H_2SO_4 acid bath and are uncorrected. Analytical TLCs were performed on pre-coated Merck silica gel $60F_{254}$ plates; the spots were detected either using UV light or by charring with 4 % alcoholic sulfuric acid. The IR spectra were recorded on a Perkin-Elmer 2000 FT-IR spectrophotometer. The optical rotations were measured with Bellingham-Stanley AD 220 polarimeter, the concentrations are expressed as g/mL. The ^{1}H-NMR and ^{13}C-NMR spectra were recorded on a Bruker Avance 300 spectrometer at 300 and 75.5 MHz, respectively in $CDCl_3$, DMSO-d_6 or CD_3CN. All the 2D measurements were performed in acetone-d_6 on a Bruker Avance 400 spectrometer. Chemical shifts are relative to internal TMS. Assignments are based on COSY, NOESY, HMBC (using Bruker’s microprogram inv4gslplrnd, which includes low-pass J-filter to suppress one-bond correlations), HSQC and JRES spectra. The chemical shift values are reported as δ ppm relative to TMS used as internal standard and the coupling constants (*J*) are measured in Hz. The ESI-HRMS spectra of all the compounds were recorded on a JEOL JMS-AX505W high-resolution mass spectrometer in positive ion mode using the matrix HEDS (*bis*-hydroxyethylsulphide) doped with sodium acetate. Acetonitrile was used after distillation over freshly ignited potassium carbonate.

2'-Deoxy-1'-(5-cyanomethyl-3-*p*-methylphenyl)pyrazolyl-3',5'-di-*O*-toluoyl-*β*-D-ribofuranose (3a)

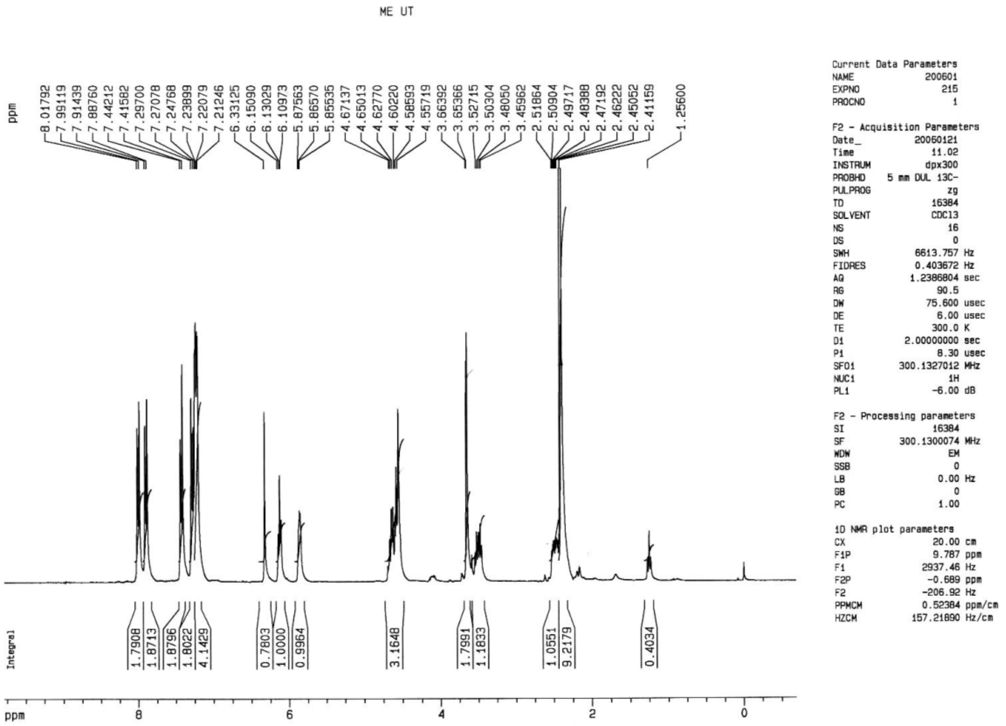

^{1}H NMR spectrum (Bruker, 300MHz, $CDCl_3$) of compound **3a**

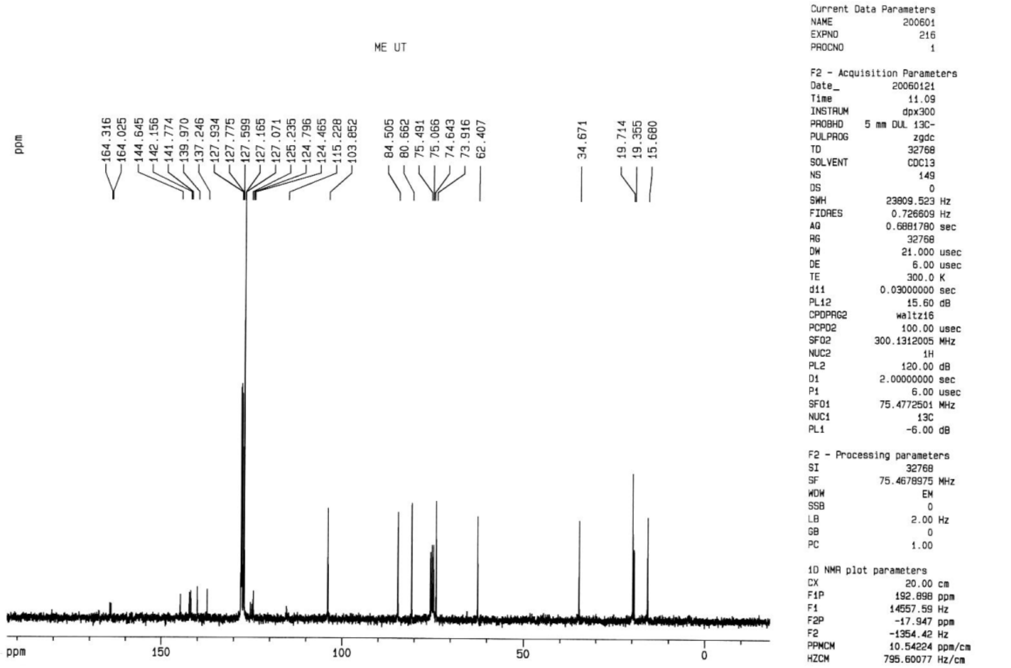

^{13}C NMR spectrum (Bruker, 75.5MHz, $CDCl_3$) of compound **3a**

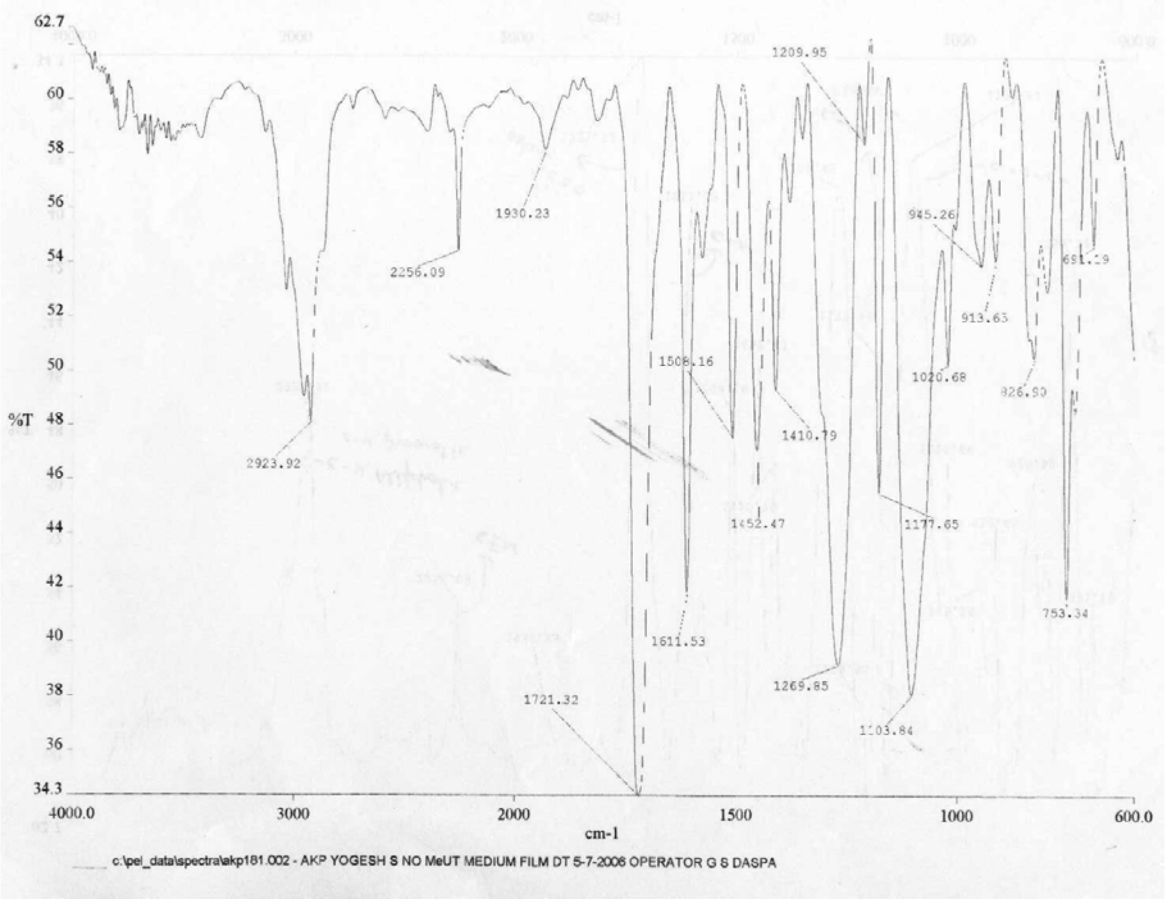

IR spectrum (Perkin-Elmer 2000 FT-IR spectrophotometer) of compound **3a**

2'-Deoxy-1'-(3-cyanomethyl-5-*p*-methoxyphenyl)pyrazolyl-3',5'-di-*O*-toluoyl-β-D-ribo furanose (3b).

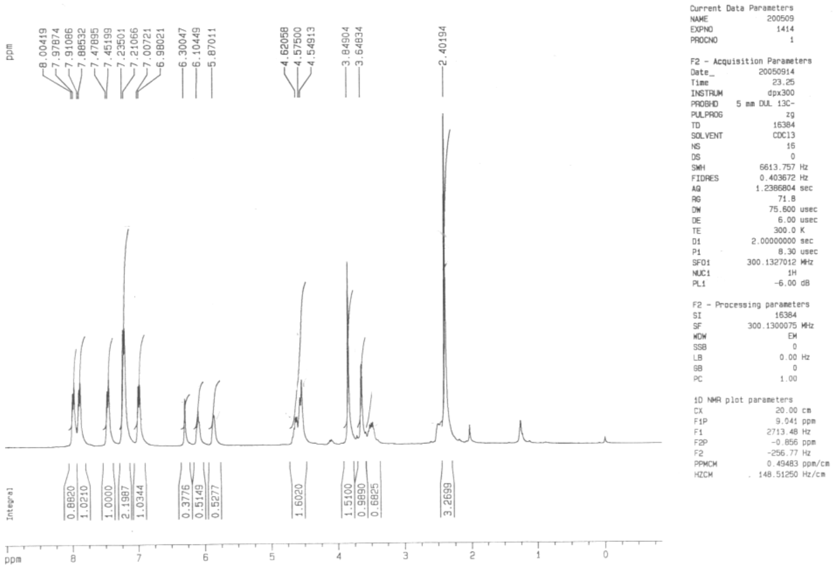

^{1}H NMR spectrum (Bruker, 300MHz, $CDCl_3$) of compound **3b**

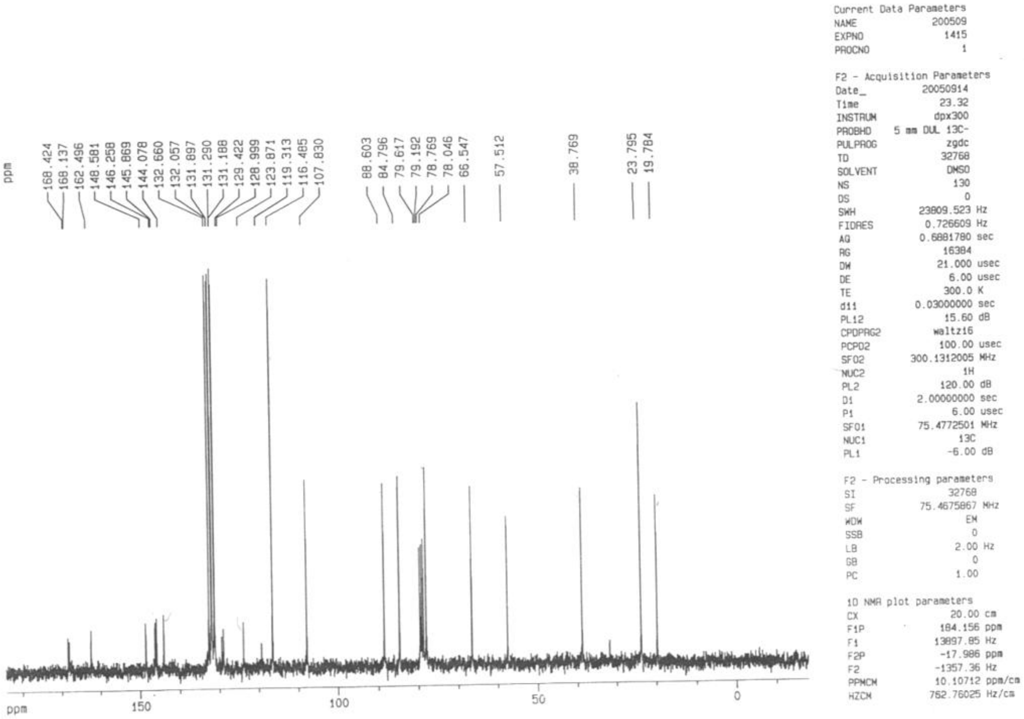

^{13}C NMR spectrum (Bruker, 75.5MHz, $CDCl_3$) of compound **3b**

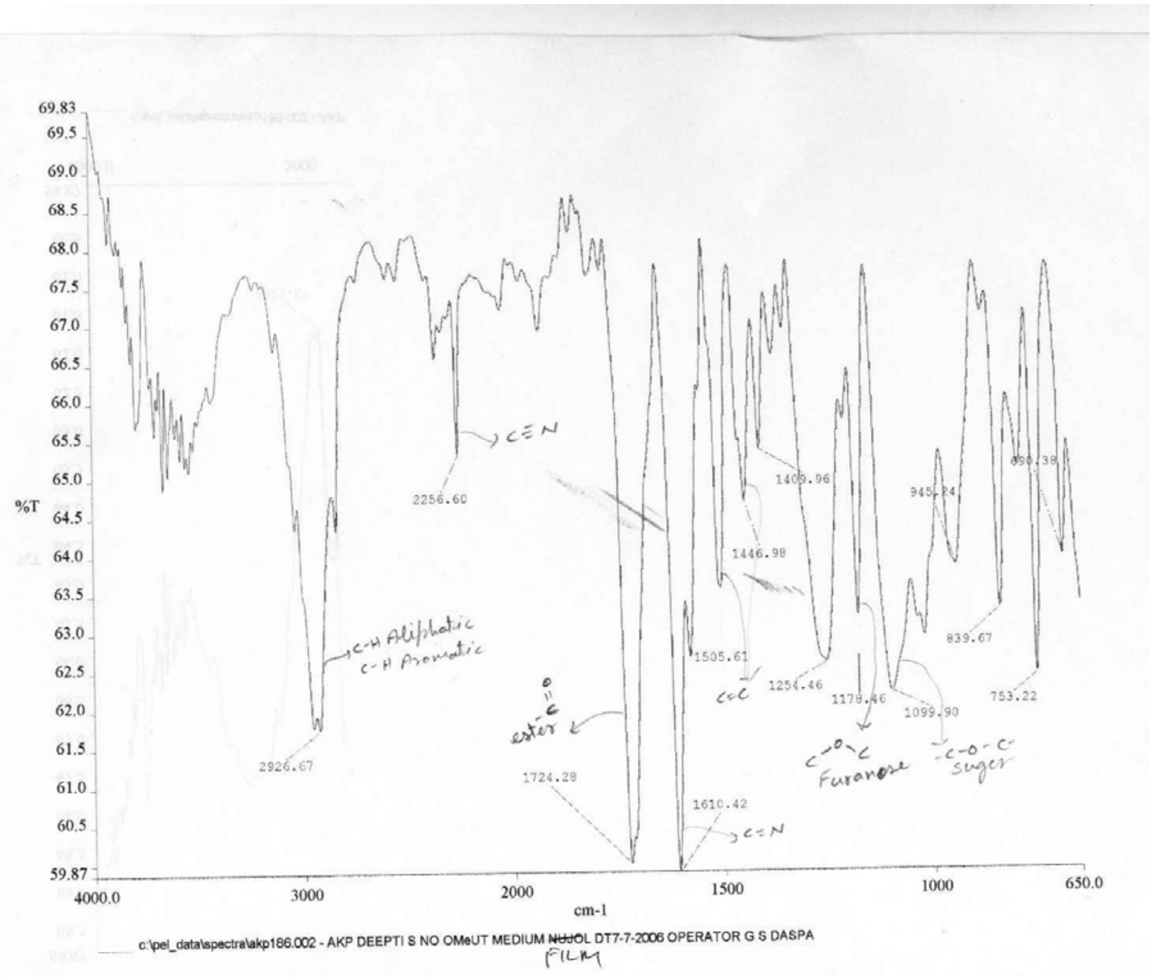

IR spectrum (Perkin-Elmer 2000 FT-IR spectrophotometer) of compound **3b**

2'-Deoxy-1'-(3-cyanomethyl-5-*p*-flurophenyl)pyrazolyl-3',5'-di-*O*-toluoyl-*β*-D-ribofuranose (3c).

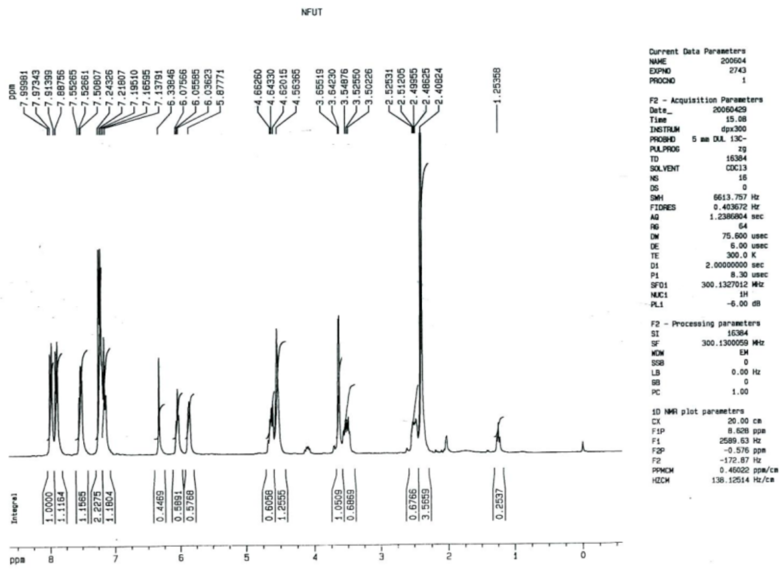

^{1}H NMR spectrum (Bruker, 300MHz, $CDCl_3$) of compound 3c

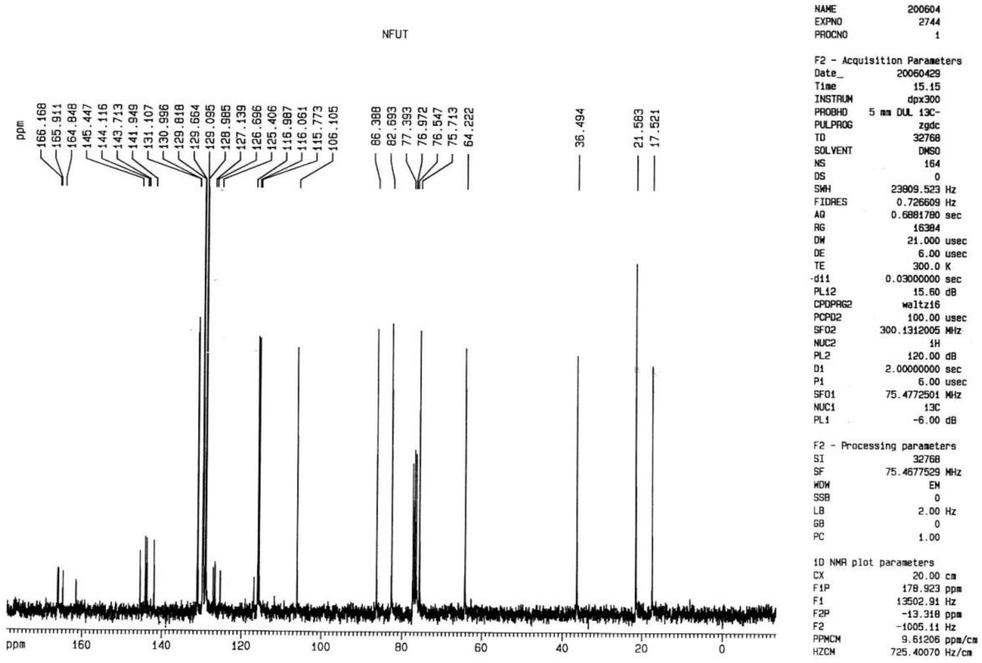

^{13}C NMR spectrum (Bruker, 75.5MHz, $CDCl_3$) of compound **3c**

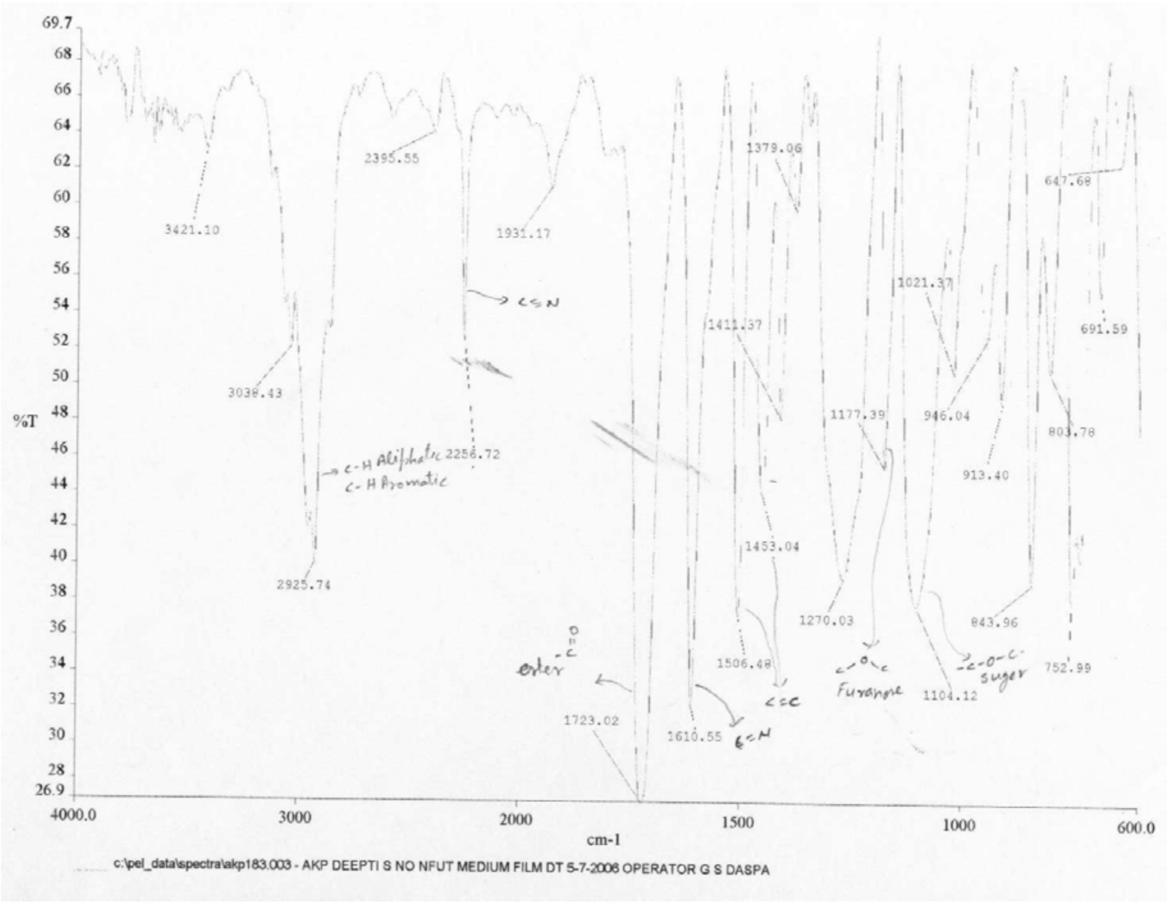

IR spectrum (Perkin-Elmer 2000 FT-IR spectrophotometer) of compound **3c**

2'-Deoxy-1'-(3-cyanomethyl-5-*p*-chlorophenyl)pyrazolyl-3',5'-di-*O*-toluoyl-*β*-D-ribofuranose (3d)

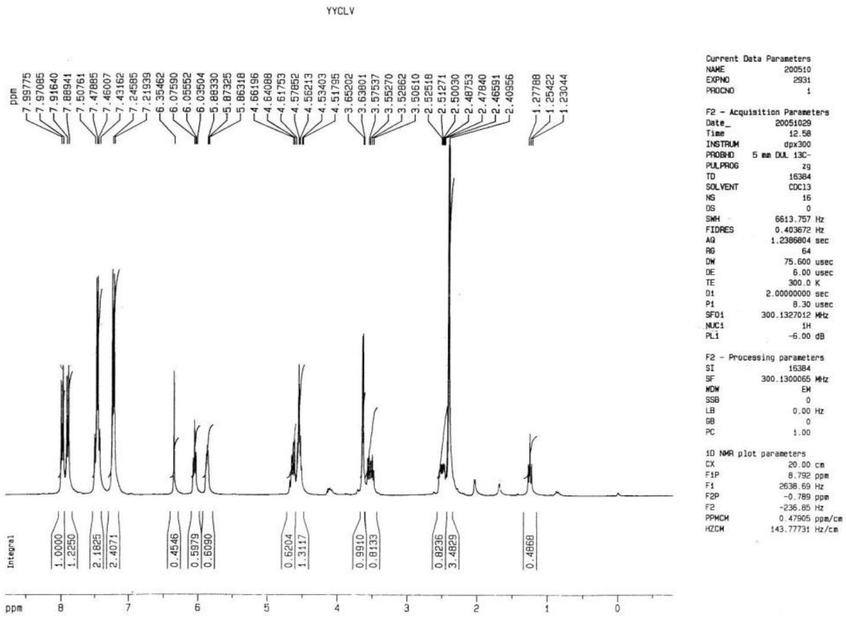

^{1}H NMR spectrum (Bruker, 300MHz, $CDCl_3$) of compound 3d

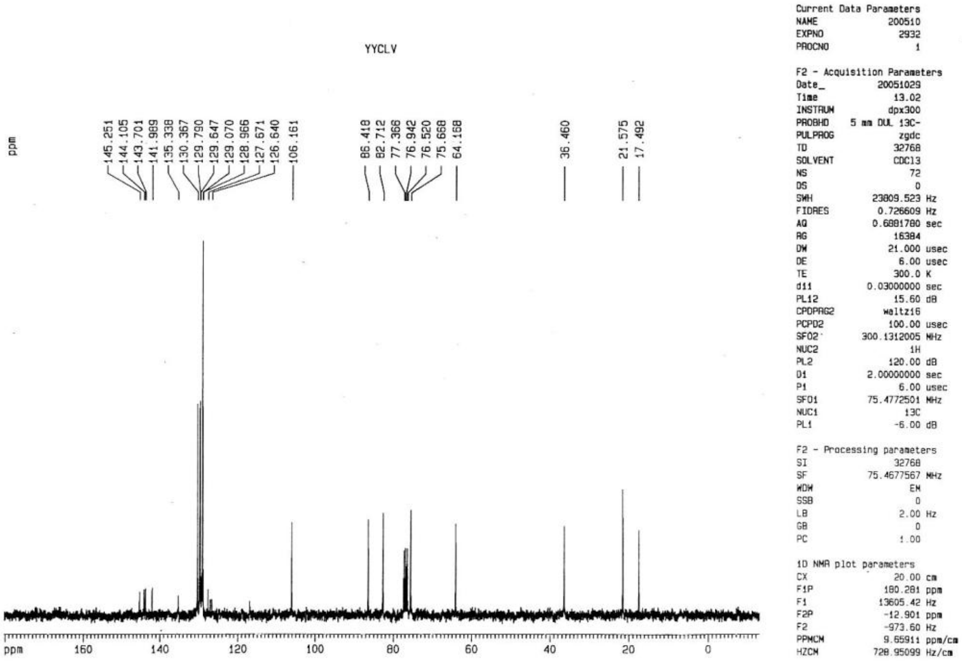

^{13}C NMR spectrum (Bruker, 75.5MHz, $CDCl_3$) of compound **3d**

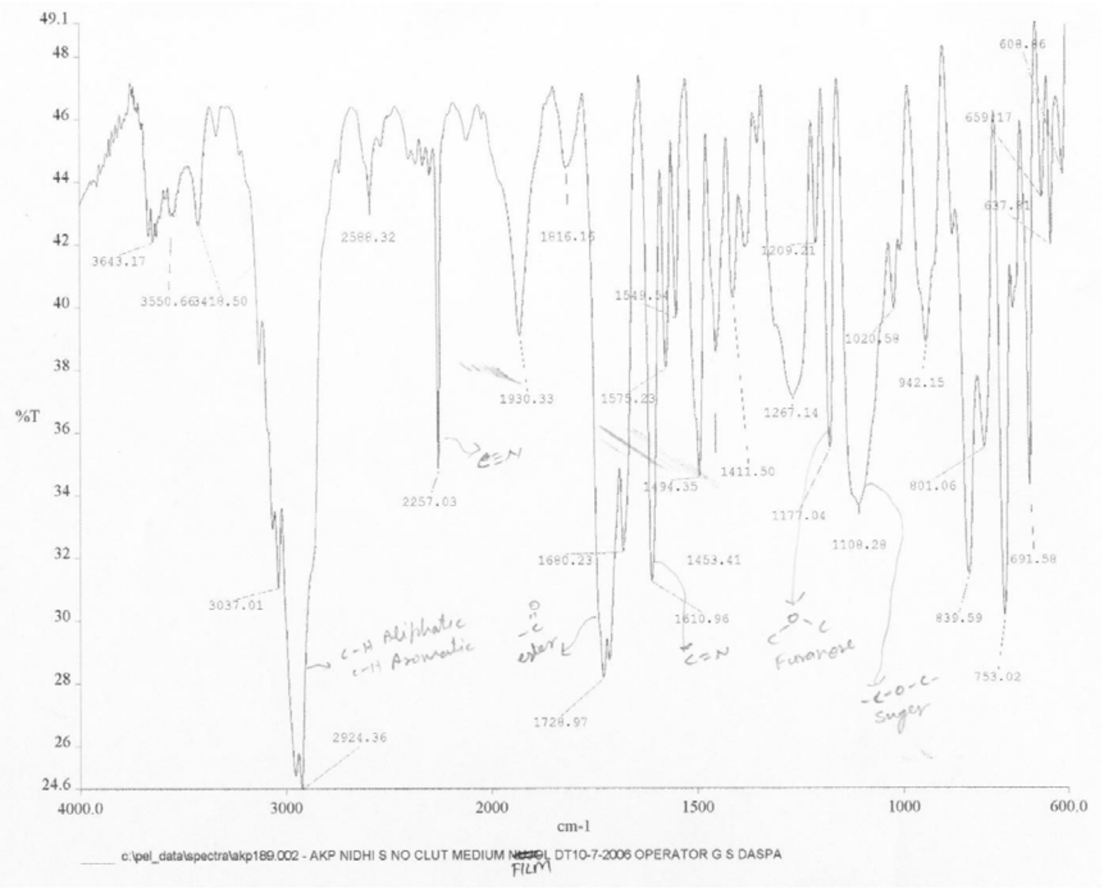

IR spectrum (Perkin-Elmer 2000 FT-IR spectrophotometer) of compound **3d**

2'-Deoxy-1'-(3-cyanomethyl-5-*p*-bromophenyl)pyrazolyl-3',5'-di-*O*-toluoyl-*β*-D-ribofuranose (3e)

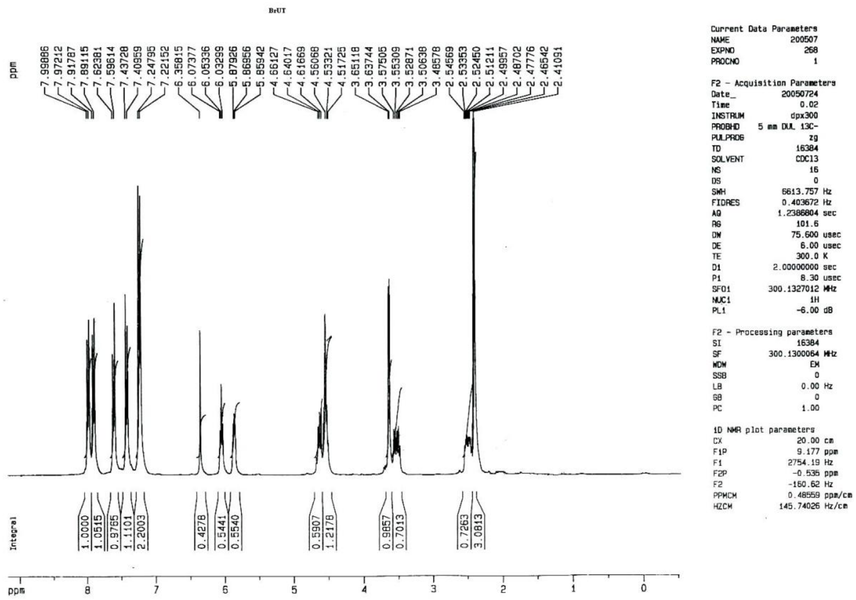

^{1}H NMR spectrum (Bruker, 300MHz, $CDCl_3$) of compound **3e**

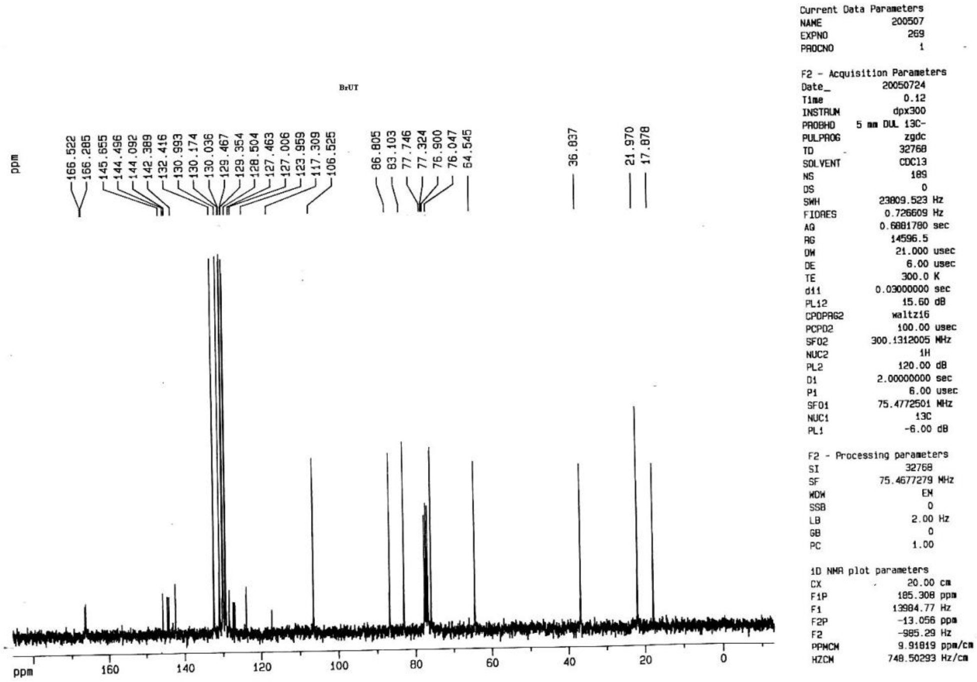

^{13}C NMR spectrum (Bruker, 75.5MHz, $CDCl_3$) of compound **3e**

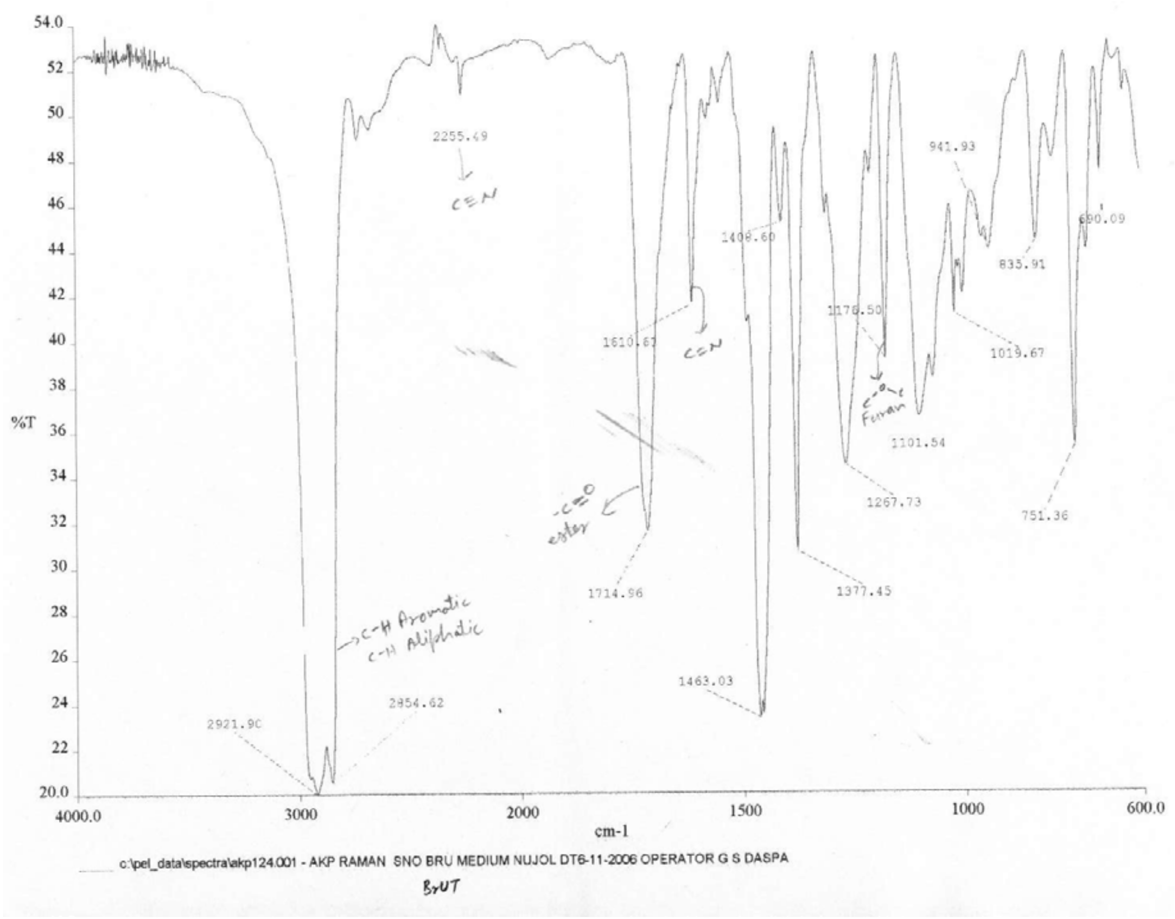

IR spectrum (Perkin-Elmer 2000 FT-IR spectrophotometer) of compound **3e**

2'-Deoxy-1'-(5-cyanomethyl-3-*p*-methylphenyl)pyrazolyl-3',5'-di-*O*-toluoyl-*β*-D-ribo furanose (4a)

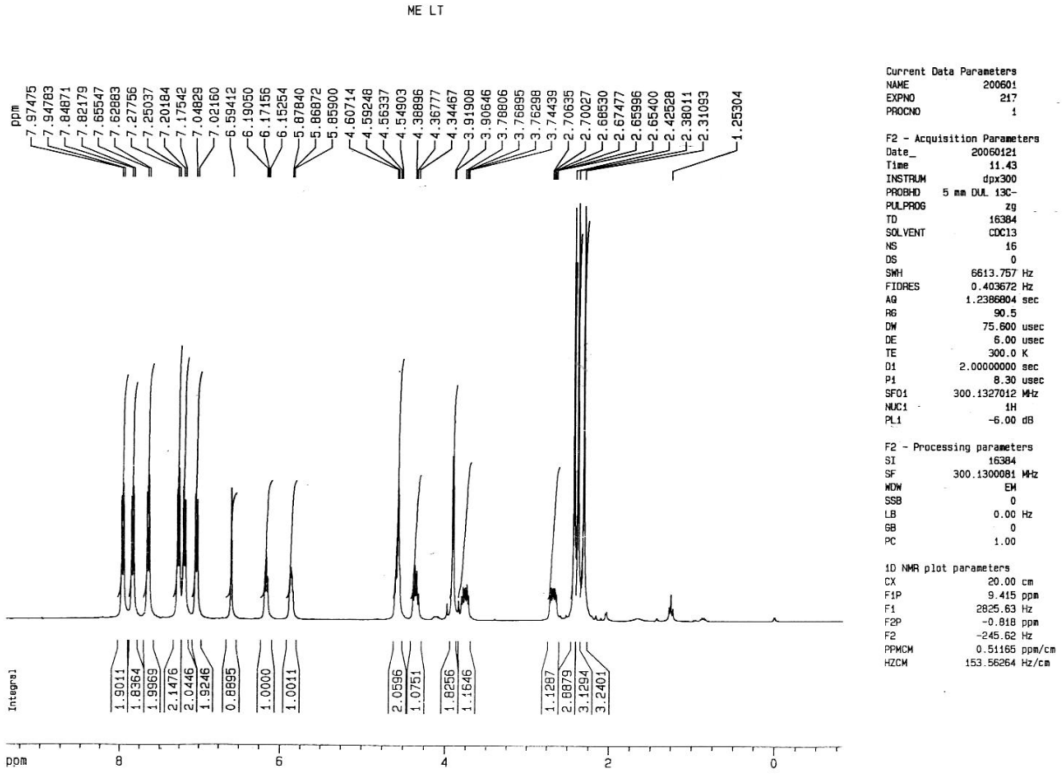

^{1}H NMR spectrum (Bruker, 300MHz, $CDCl_3$) of compound **4a**

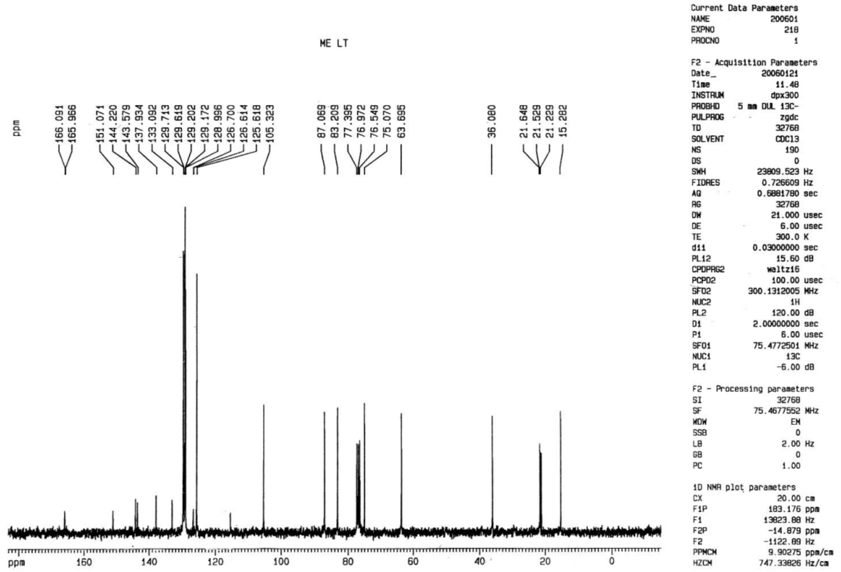

^{13}C NMR spectrum (Bruker, 75.5MHz, $CDCl_3$) of compound **4a**

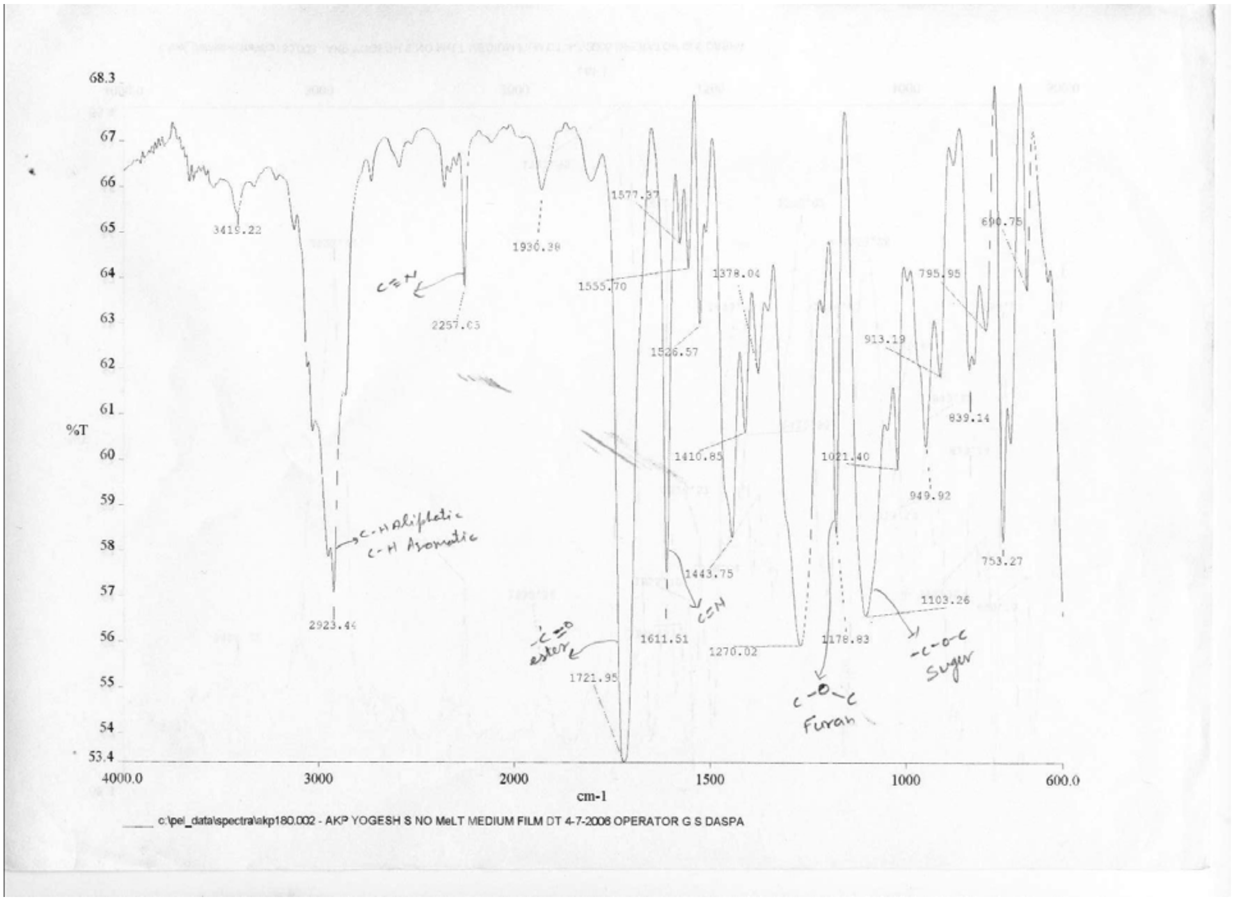

IR spectrum (Perkin-Elmer 2000 FT-IR spectrophotometer) of compound **4a**

2'-deoxy-1'-(5-cyanomethyl-3-*p*-methoxyphenyl)pyrazolyl-3',5'-di-*O*-toluoyl-*β*-D-ribofuranose (4b).

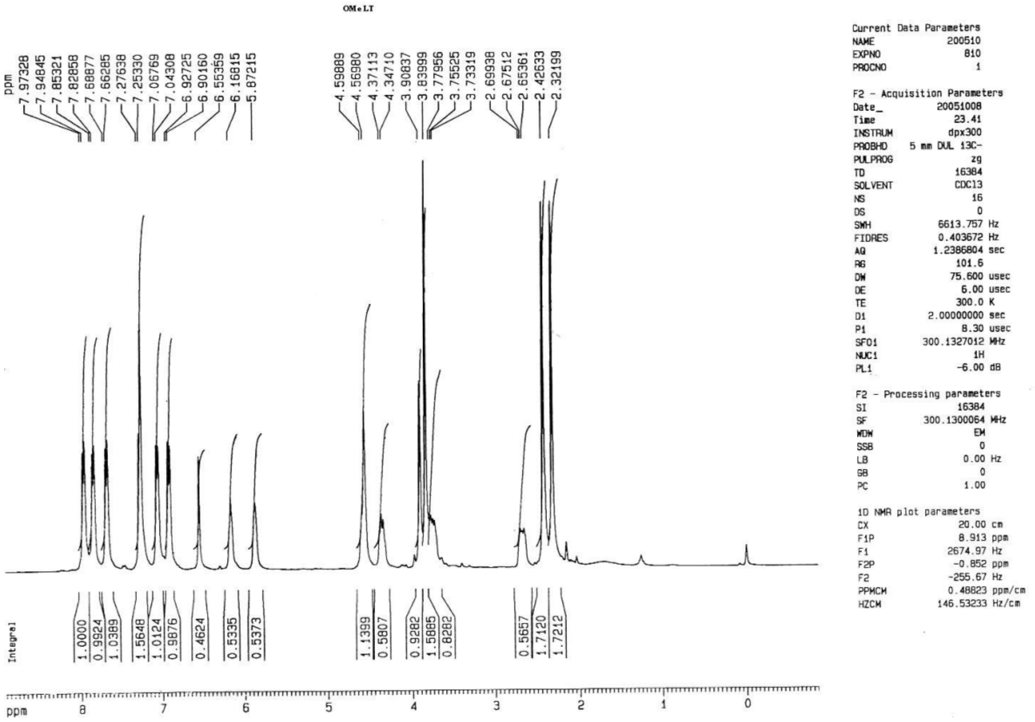

^{1}H NMR spectrum (Bruker, 300MHz, $CDCl_3$) of compound **4b**

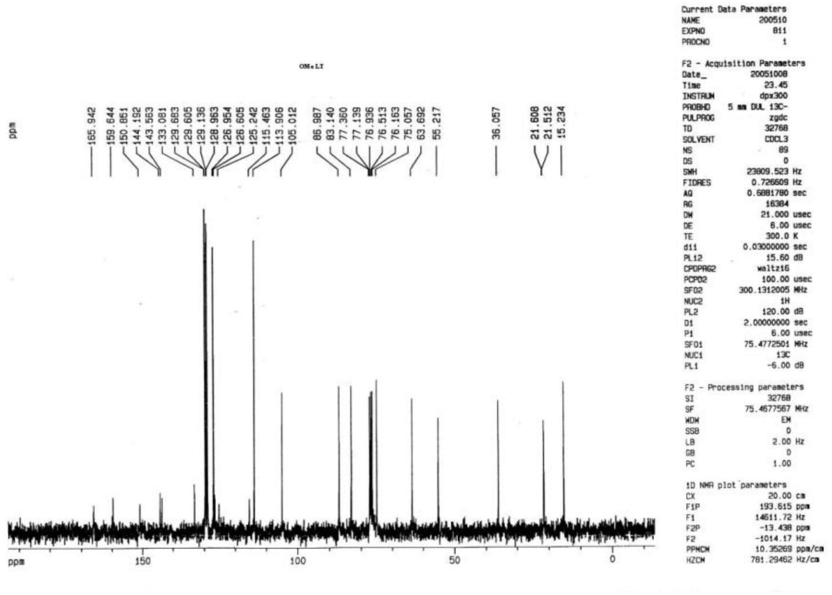

^{13}C NMR spectrum (Bruker, 75.5MHz, $CDCl_3$) of compound **4b**

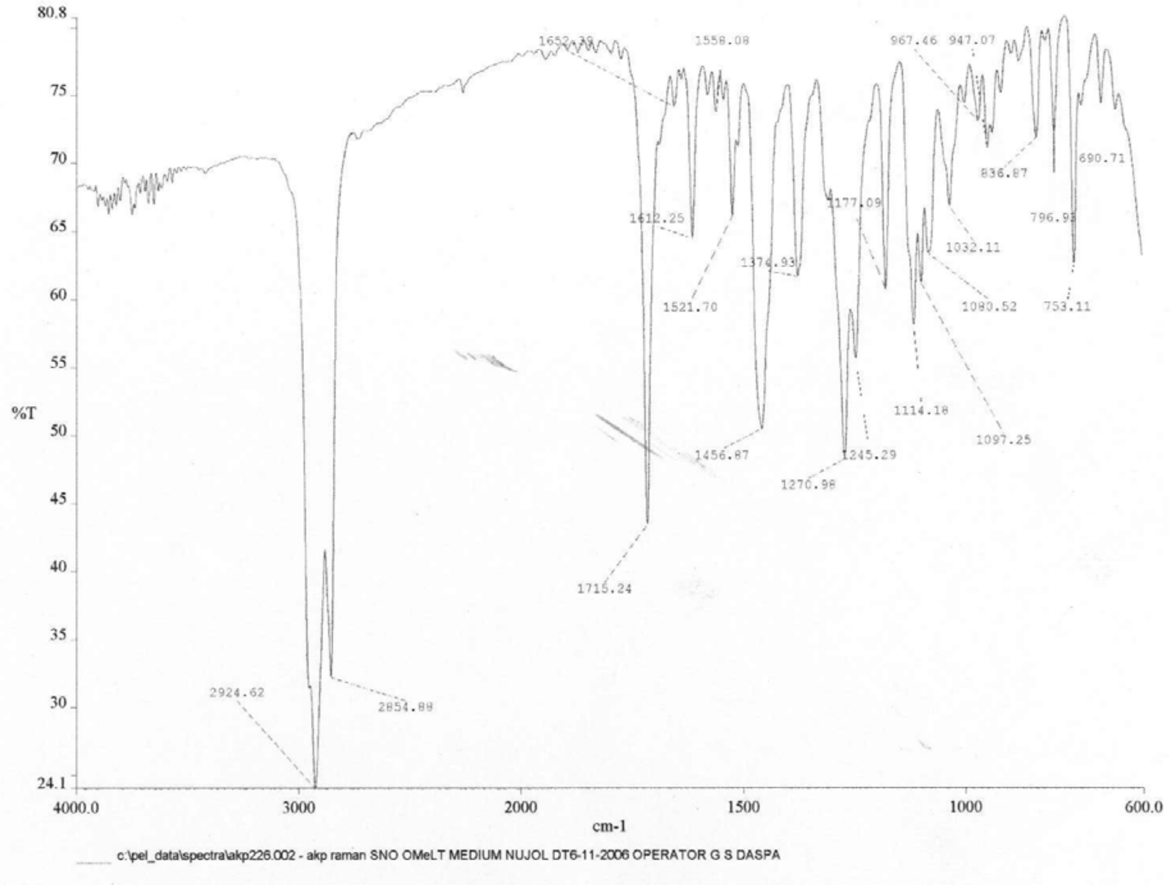

IR spectrum (Perkin-Elmer 2000 FT-IR spectrophotometer) of compound **4b**

2'-Deoxy-1'-(5-cyanomethyl-3-*p*-flurophenyl)pyrazolyl-3',5'-di-*O*-toluoyl-*β*-D-ribofuranose (4c).

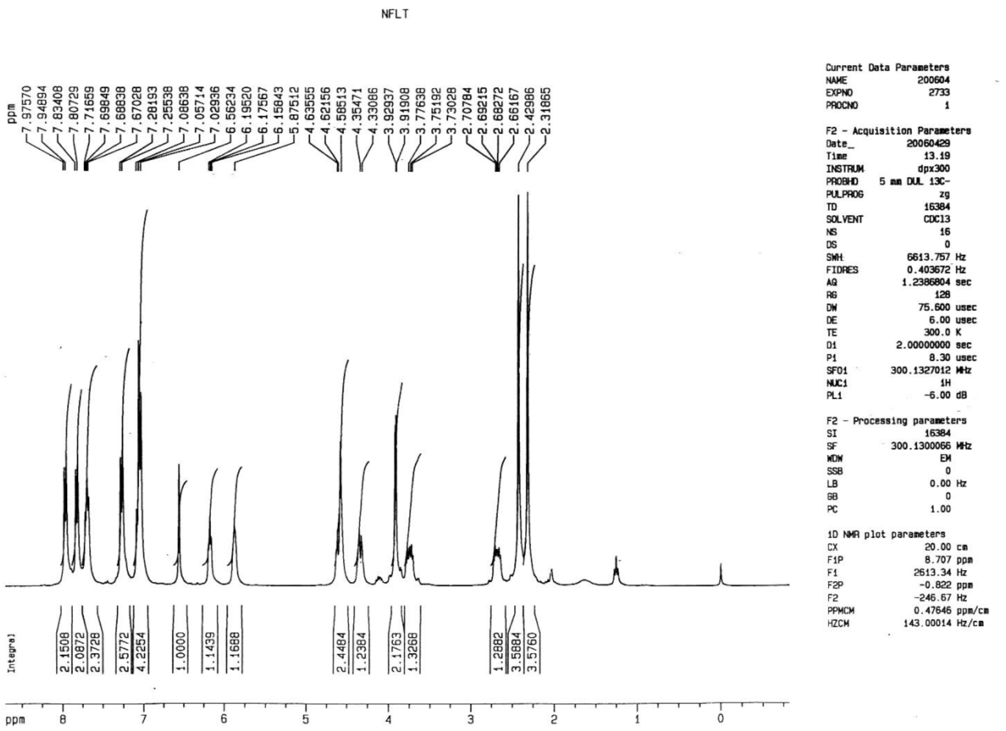

^{1}H NMR spectrum (Bruker, 300MHz, $CDCl_3$) of compound **4c**

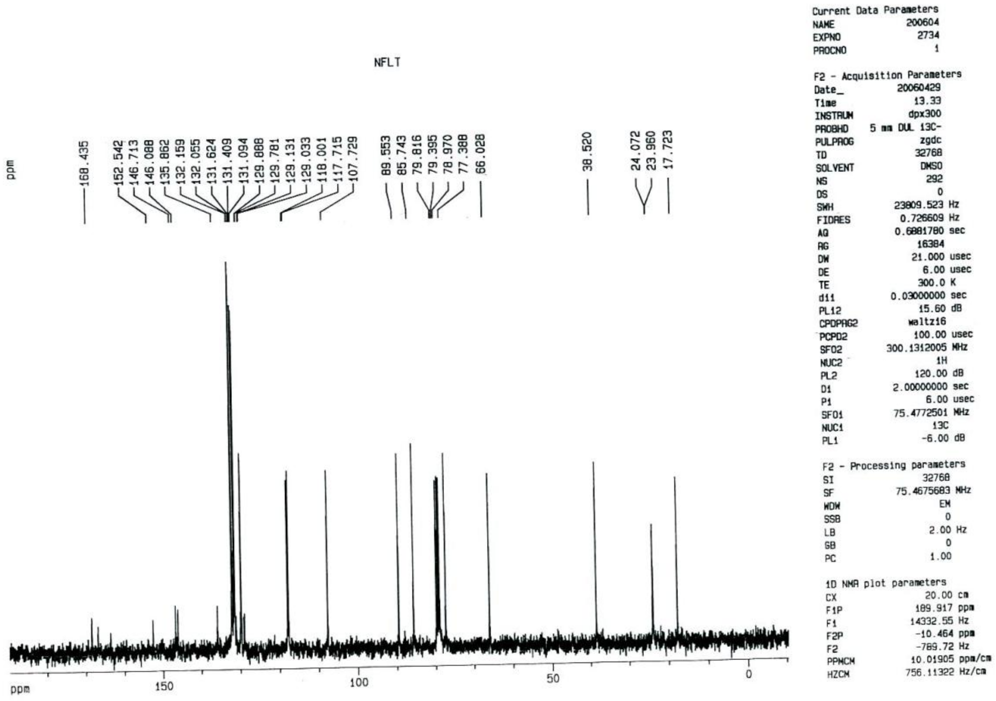

^{13}C NMR spectrum (Bruker, 75.5MHz, $CDCl_3$) of compound **4c**

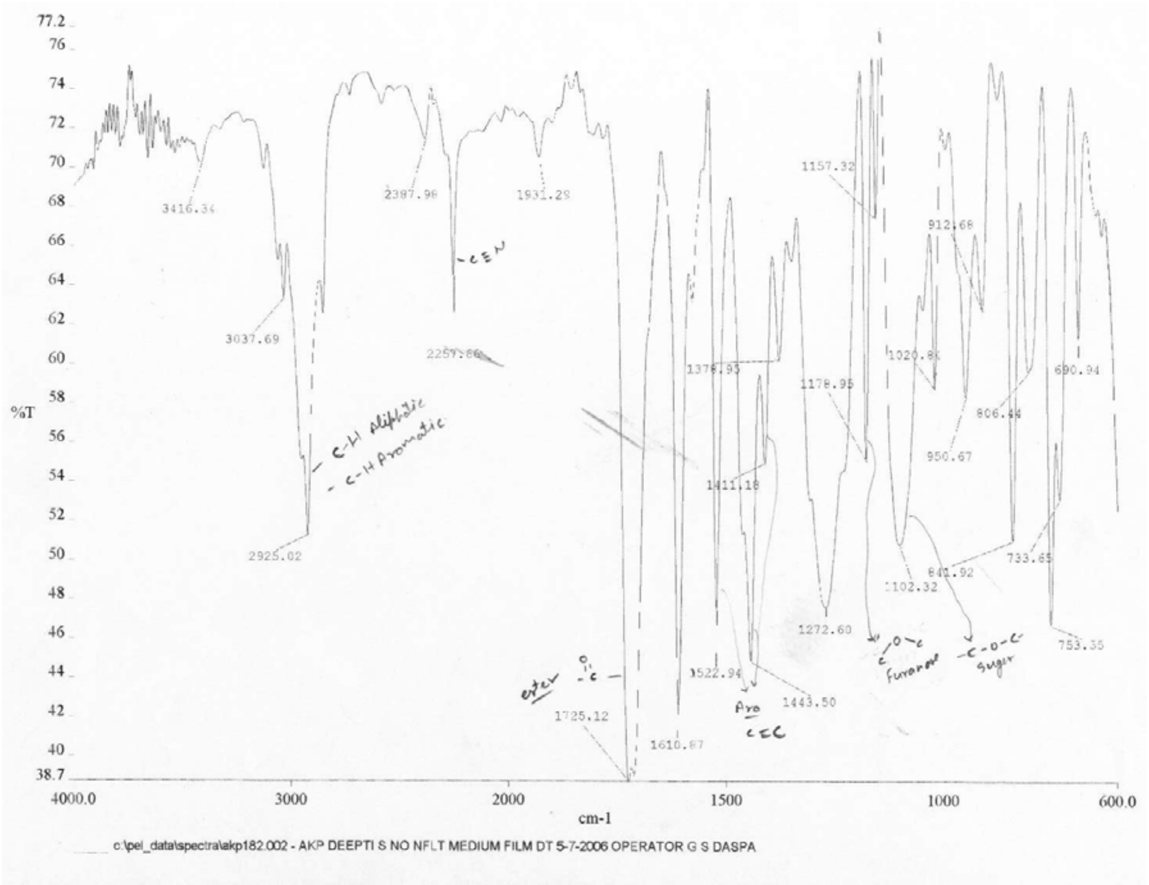

IR spectrum (Perkin-Elmer 2000 FT-IR spectrophotometer) of compound **4c**

2'-Deoxy-1'-(5-cyanomethyl-3-*p*-chlorophenyl)pyrazolyl-3',5'-di-*O*-toluoyl-*β*-D-ribofuranose (4d).

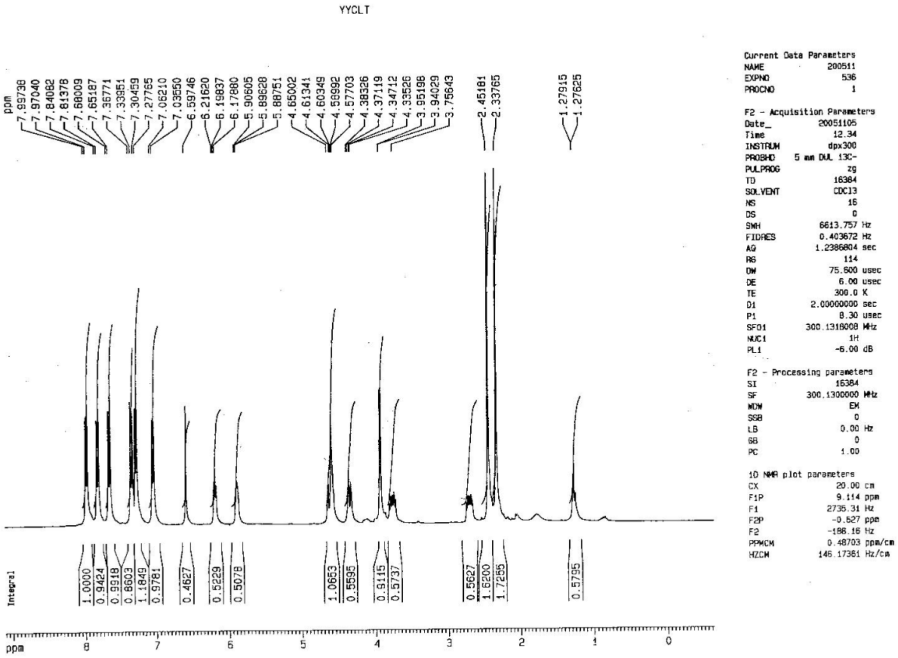

^{1}H NMR spectrum (Bruker, 300MHz, $CDCl_3$) of compound **4d**

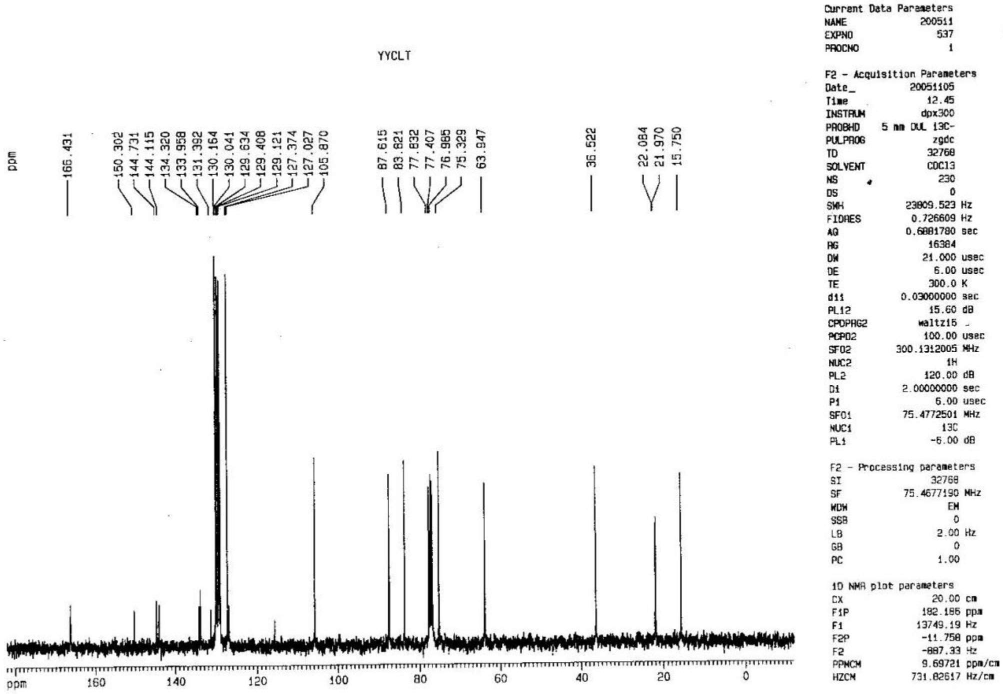

^{13}C NMR spectrum (Bruker, 75.5MHz, $CDCl_3$) of compound **4d**

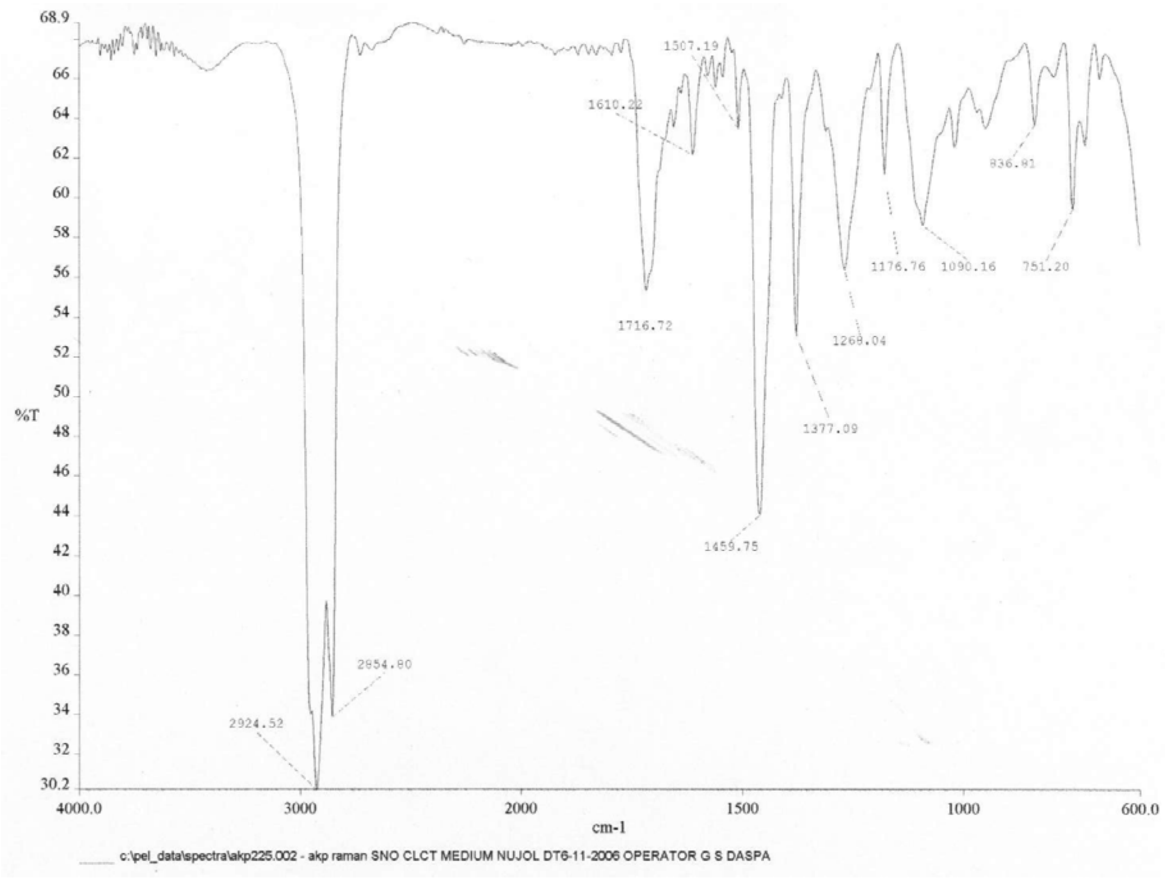

IR spectrum (Perkin-Elmer 2000 FT-IR spectrophotometer) of compound **4d**

2'-Deoxy-1'-(5-cyanomethyl-3-*p*-bromophenyl)pyrazolyl-3',5'-di-*O*-toluoyl-*β*-D-ribofuranose (4e)

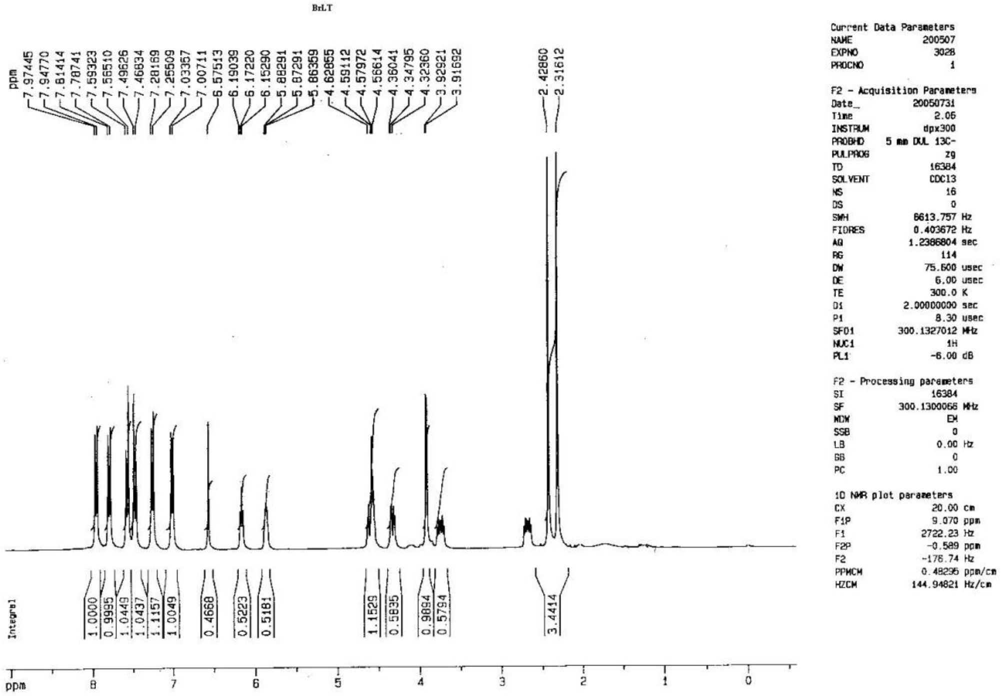

^{1}H NMR spectrum (Bruker, 300MHz, $CDCl_3$) of compound **4e**

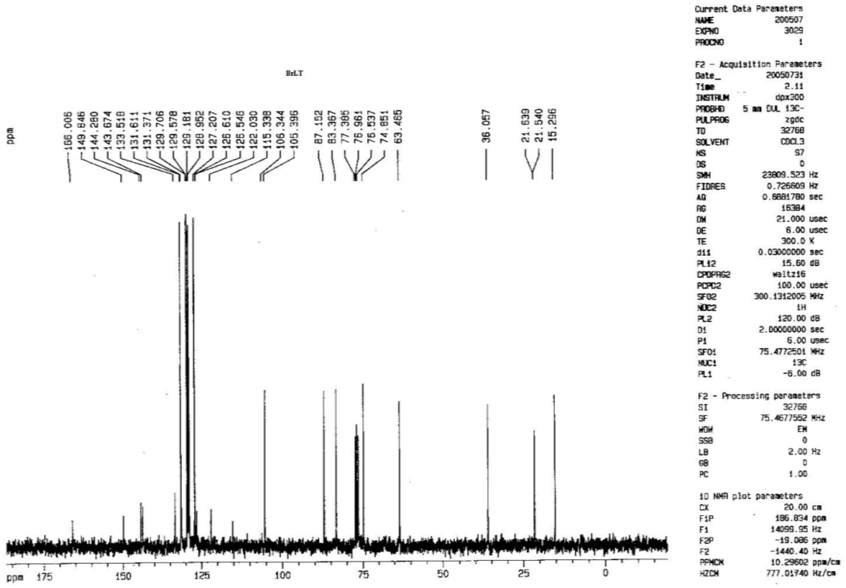

^{13}C NMR spectrum (Bruker, 75.5MHz, $CDCl_3$) of compound **4e**

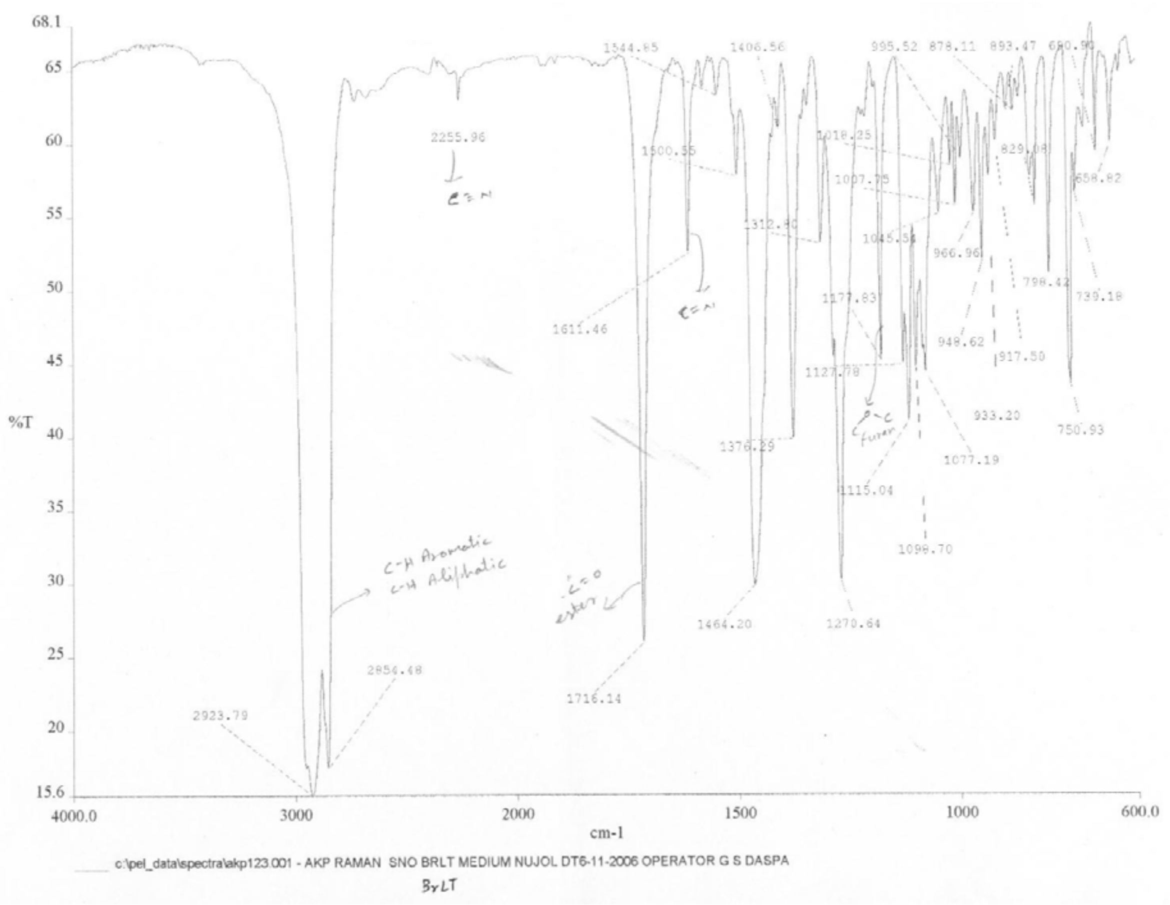

IR spectrum (Perkin-Elmer 2000 FT-IR spectrophotometer) of compound **4e**

2'-Deoxy-1'-(3-cyanomethyl-5-*p*-methylphenyl)pyrazolyl-*β*-D-ribofuranose (5a).

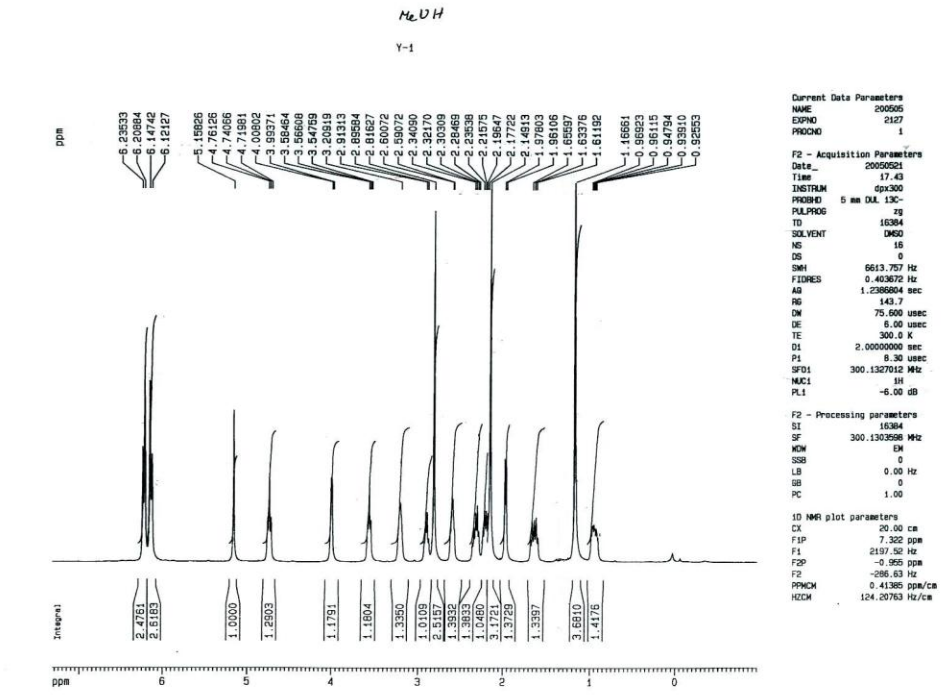

^{1}H NMR spectrum (Bruker, 300MHz, DMSO) of compound 5a

2'-Deoxy-1'-(3-cyanomethyl-5-*p*-methoxyphenyl)pyrazolyl-*β*-D-ribofuranose (5b).

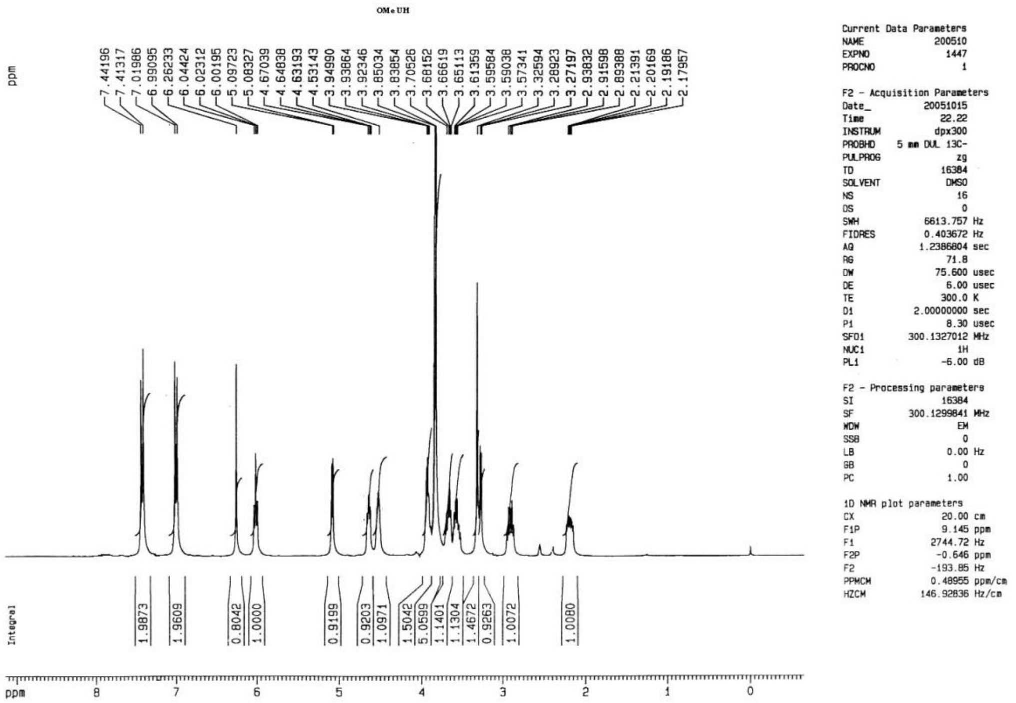

^{1}H NMR spectrum (Bruker, 300MHz, mixture of $CDCl_3$ & DMSO) of compound **5b**

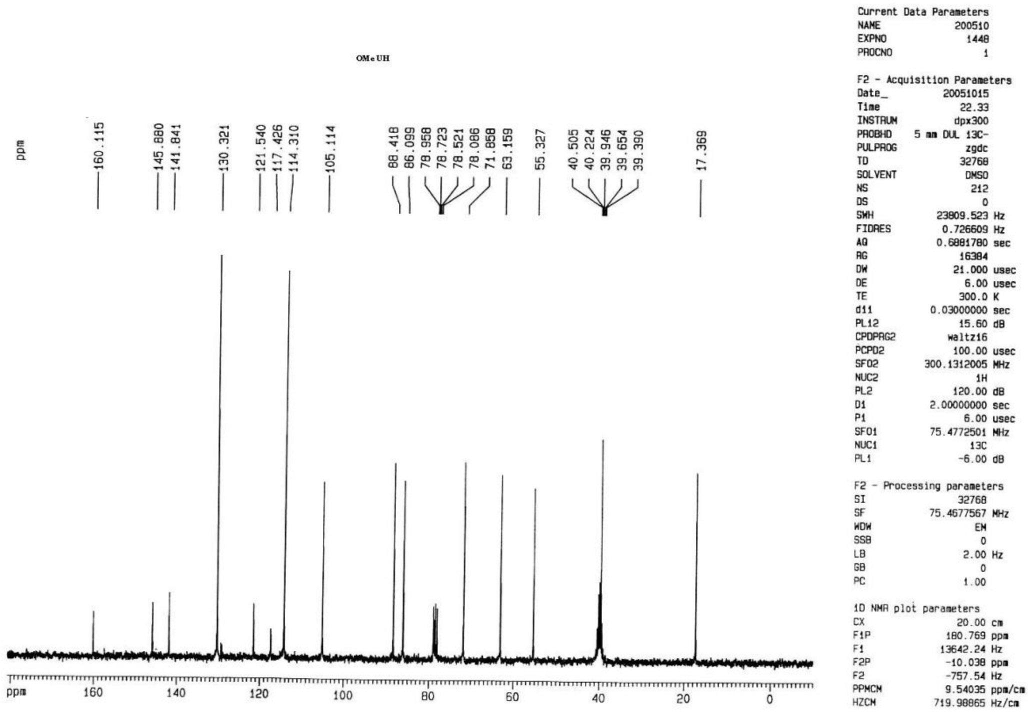

^{13}C NMR spectrum (Bruker, 75.5MHz, mixture of $CDCl_3$ & DMSO) of compound **5b**

2'-Deoxy-1'-(3-cyanomethyl-5-*p*-fluorophenyl)pyrazolyl-*β*-D-ribofuranose (5c).

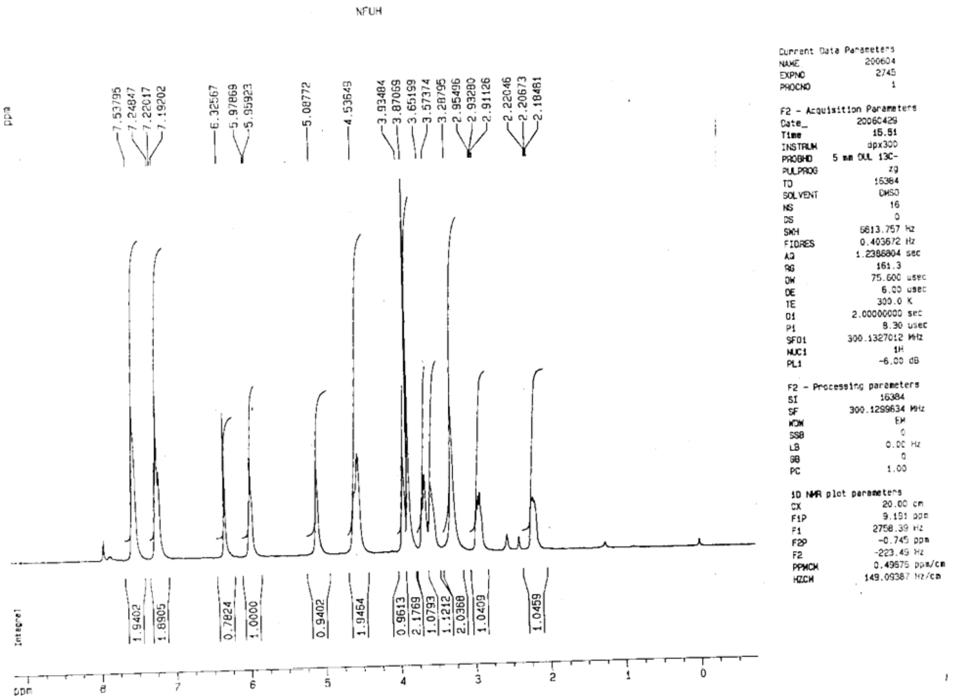

^{1}H NMR spectrum (Bruker, 300MHz, mixture of $CDCl_3$ & DMSO) of compound **5c**

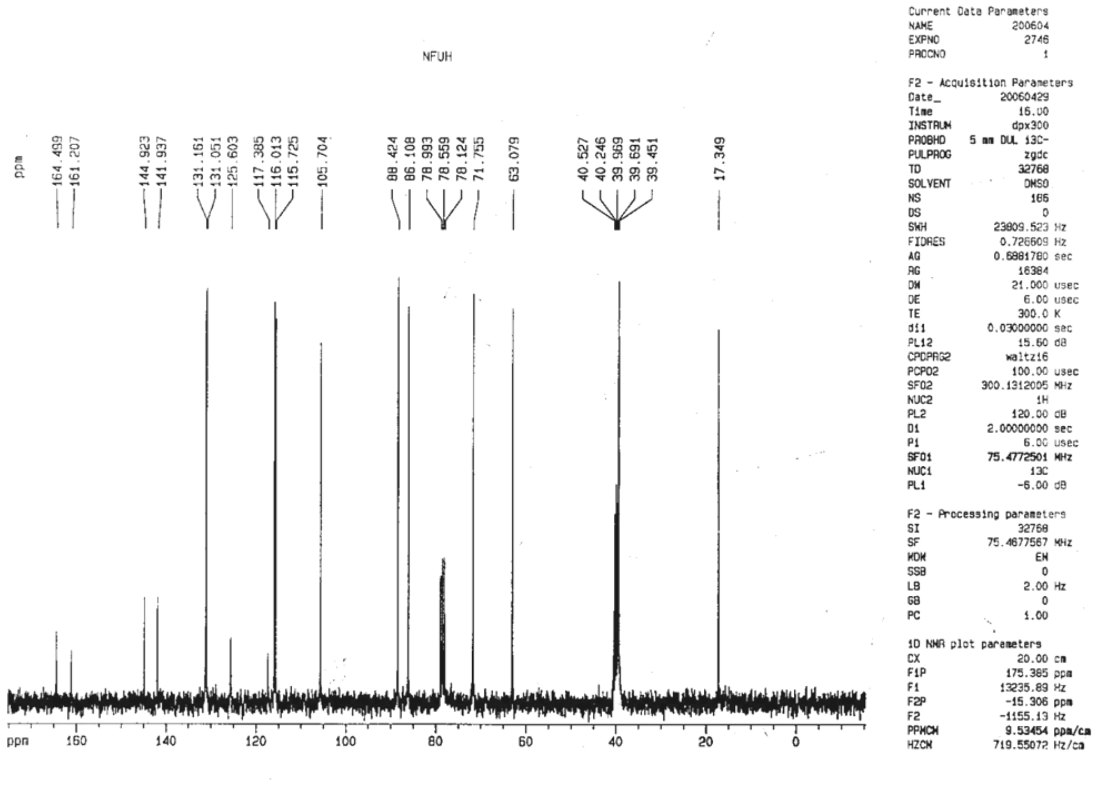

^{13}C NMR spectrum (Bruker, 75.5MHz, mixture of $CDCl_3$ & DMSO) of compound **5c**

2'-Deoxy-1'-(3-cyanomethyl-5-*p*-chlorophenyl)pyrazolyl-*β*-D-ribofuranose (5d)

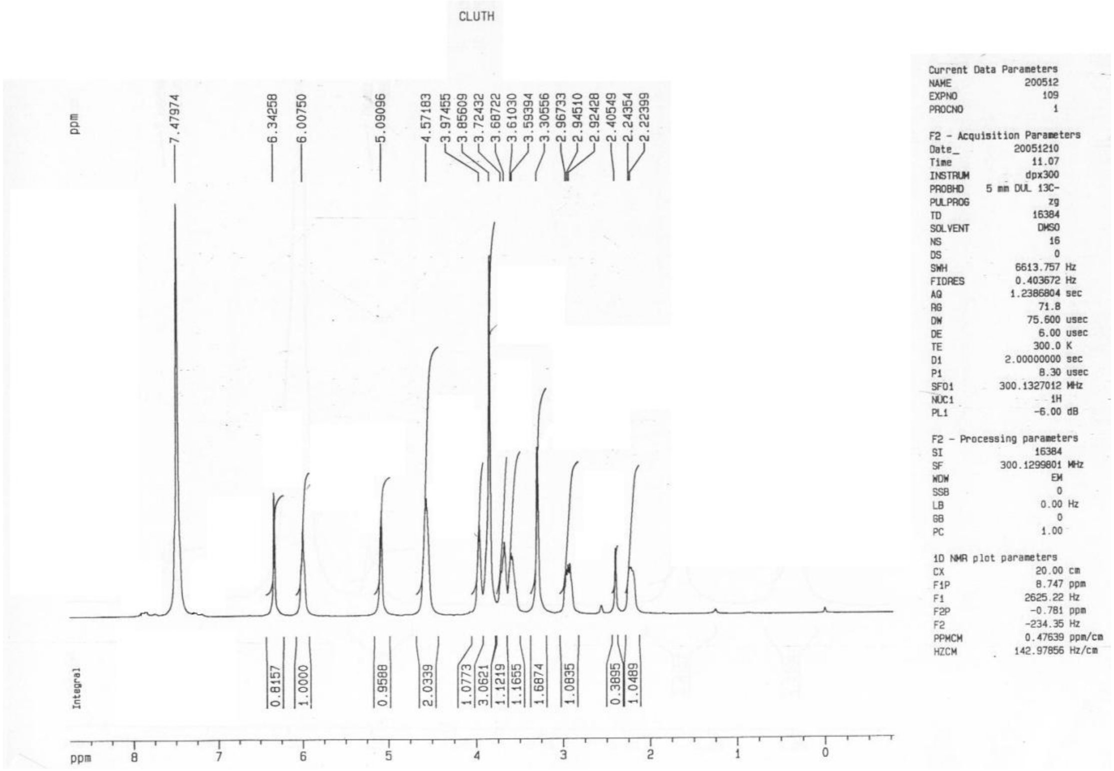

^{1}H NMR spectrum (Bruker, 300MHz, mixture of $CDCl_3$ & DMSO) of compound **5d**

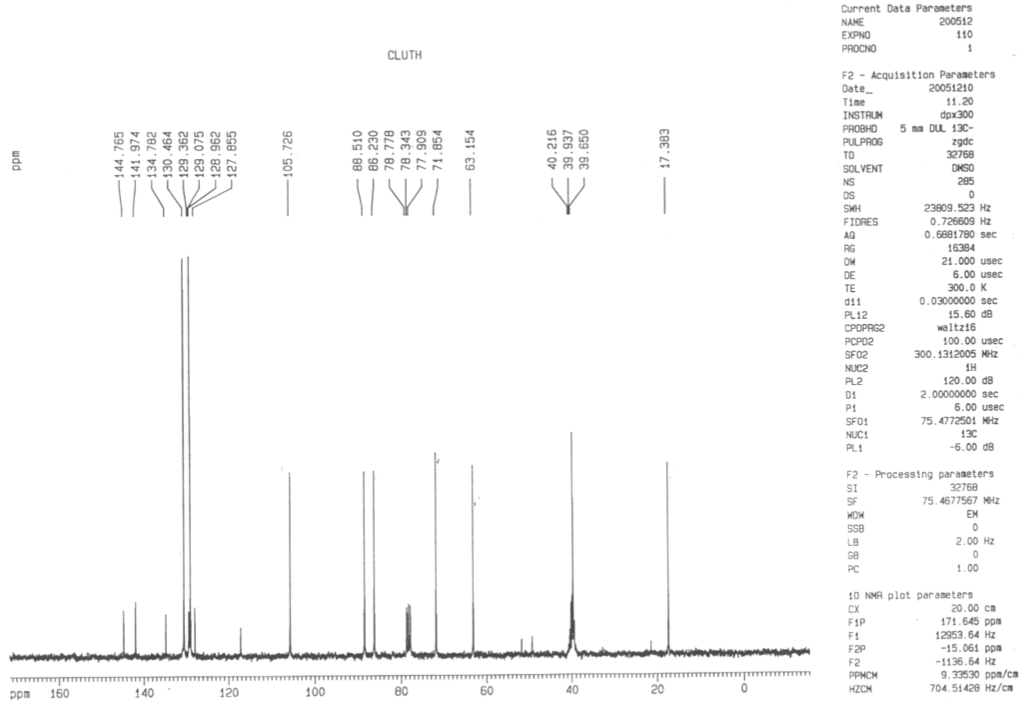

^{13}C NMR spectrum (Bruker, 75.5MHz, mixture of $CDCl_3$ & DMSO) of compound **5d**

2'-Deoxy-1'-(3-cyanomethyl-5-*p*-bromophenyl)pyrazolyl-*β*-D-ribofuranose (5e).

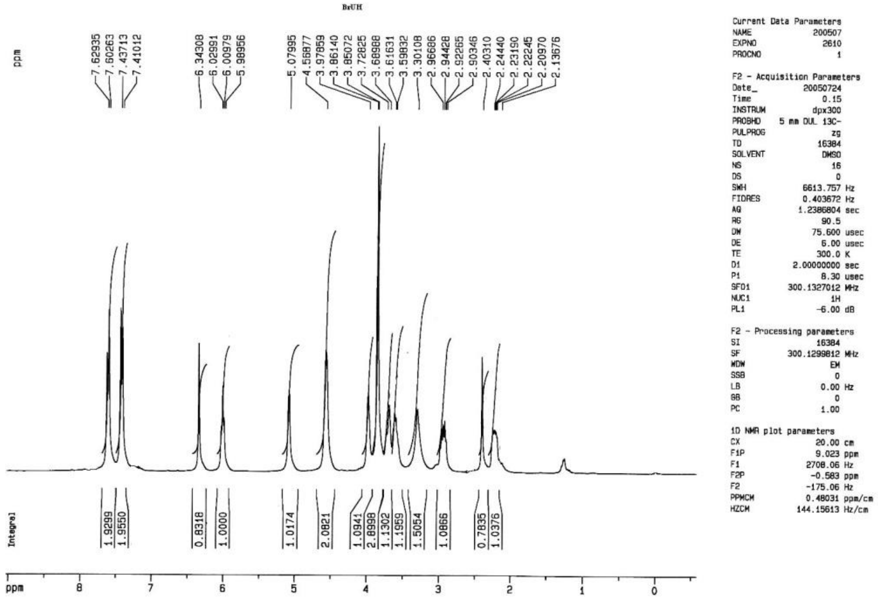

^{1}H NMR spectrum (Bruker, 300MHz, mixture of $CDCl_3$ & DMSO) of compound **5e**

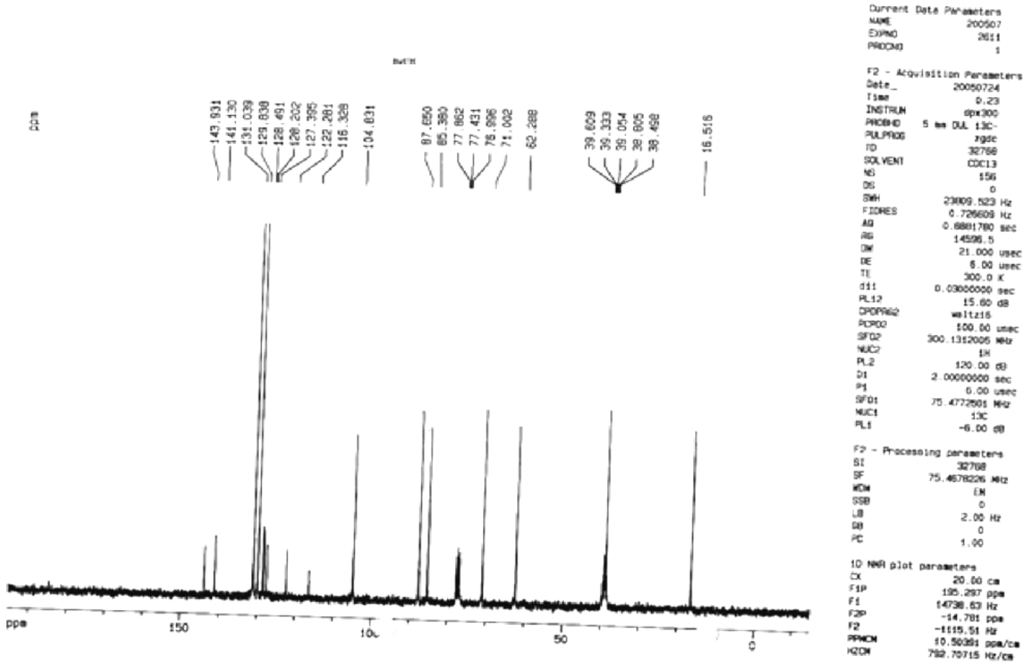

^{13}C NMR spectrum (Bruker, 75.5MHz, mixture of $CDCl_3$ & DMSO) of compound **5d**

2'-Deoxy-1'-(5-cyanomethyl-3-*p*-methylphenyl)pyrazolyl-β-D-ribofuranose (6a)

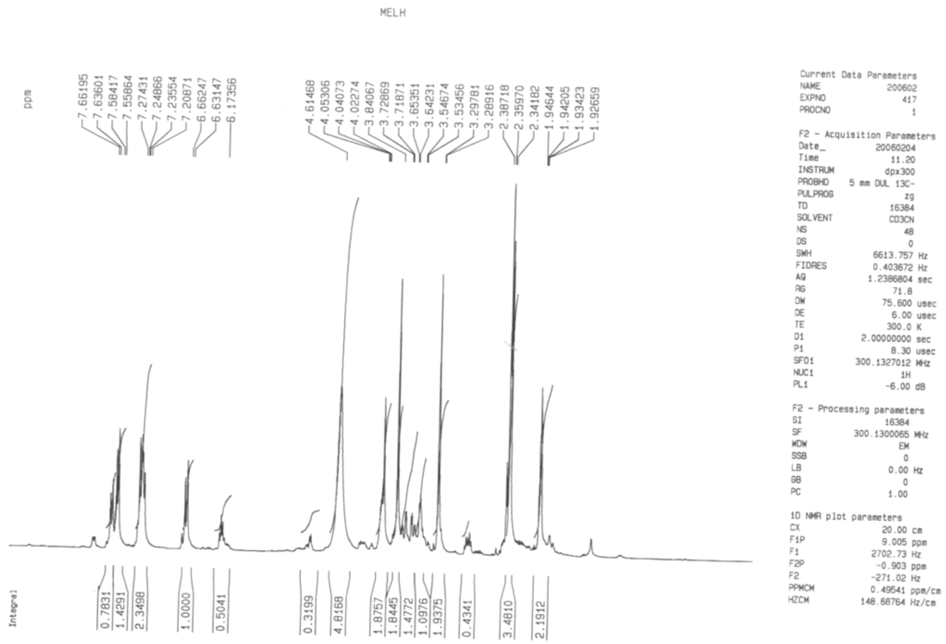

^{1}H NMR spectrum (Bruker, 300MHz, mixture of CD_3CN) of compound **6a**

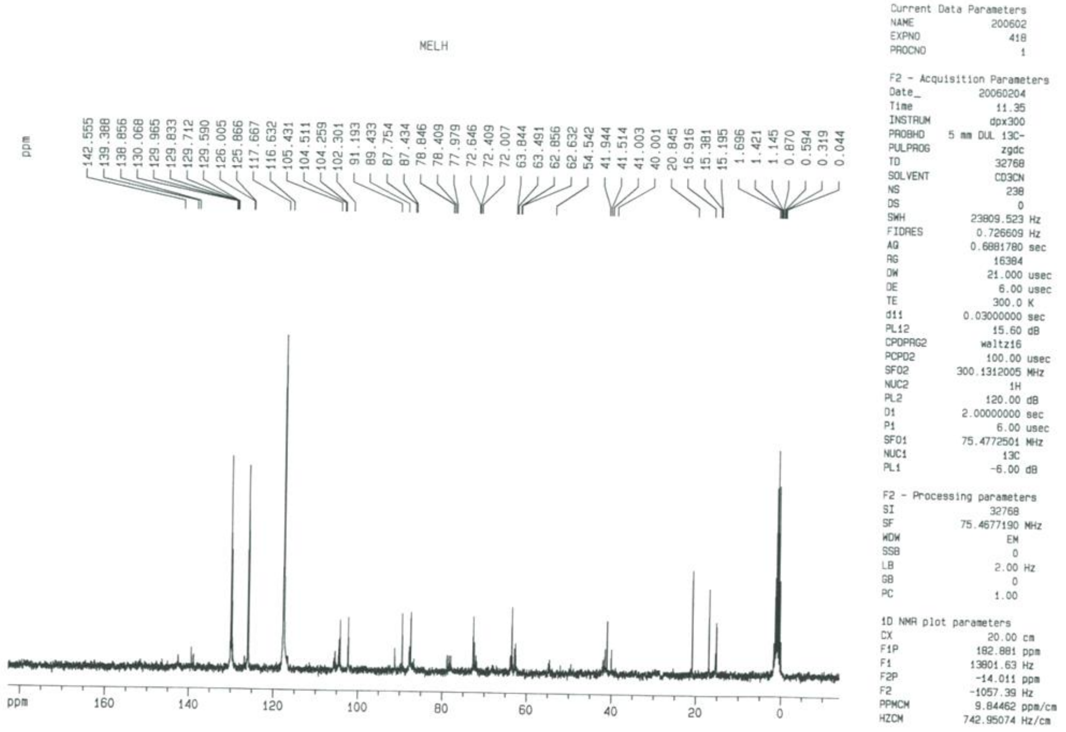

^{13}C NMR spectrum (Bruker, 75.5MHz, mixture of CD_3CN) of compound **6a**

2'-Deoxy-1'-(3-cyanomethyl-5-*p*-methoxyphenyl)pyrazolyl-*β*-D-ribofuranose (6b)

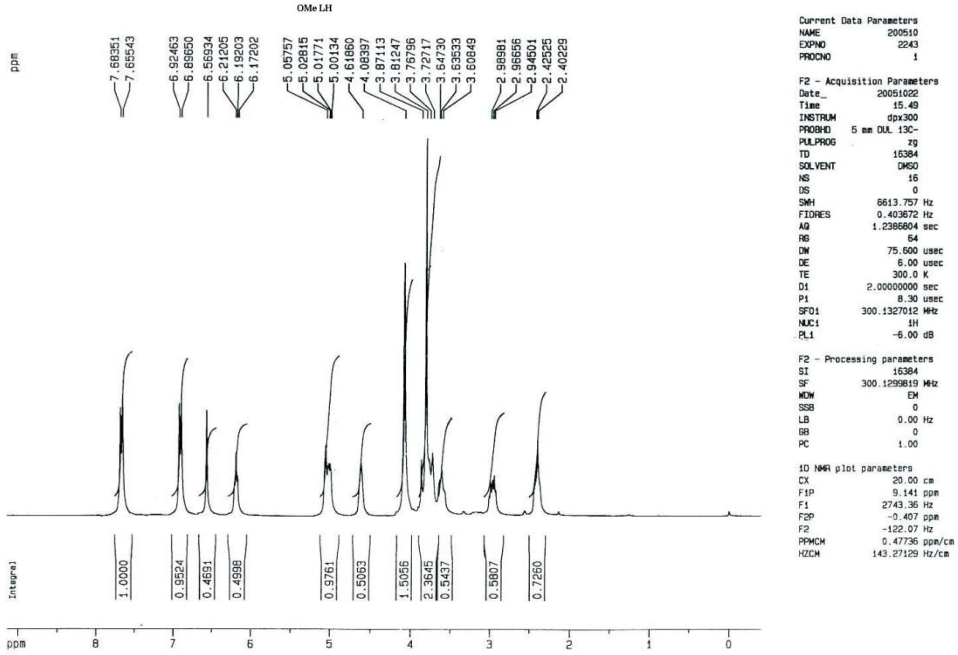

^{1}H NMR spectrum (Bruker, 300MHz, mixture of $CDCl_3$ & DMSO) of compound **6b**

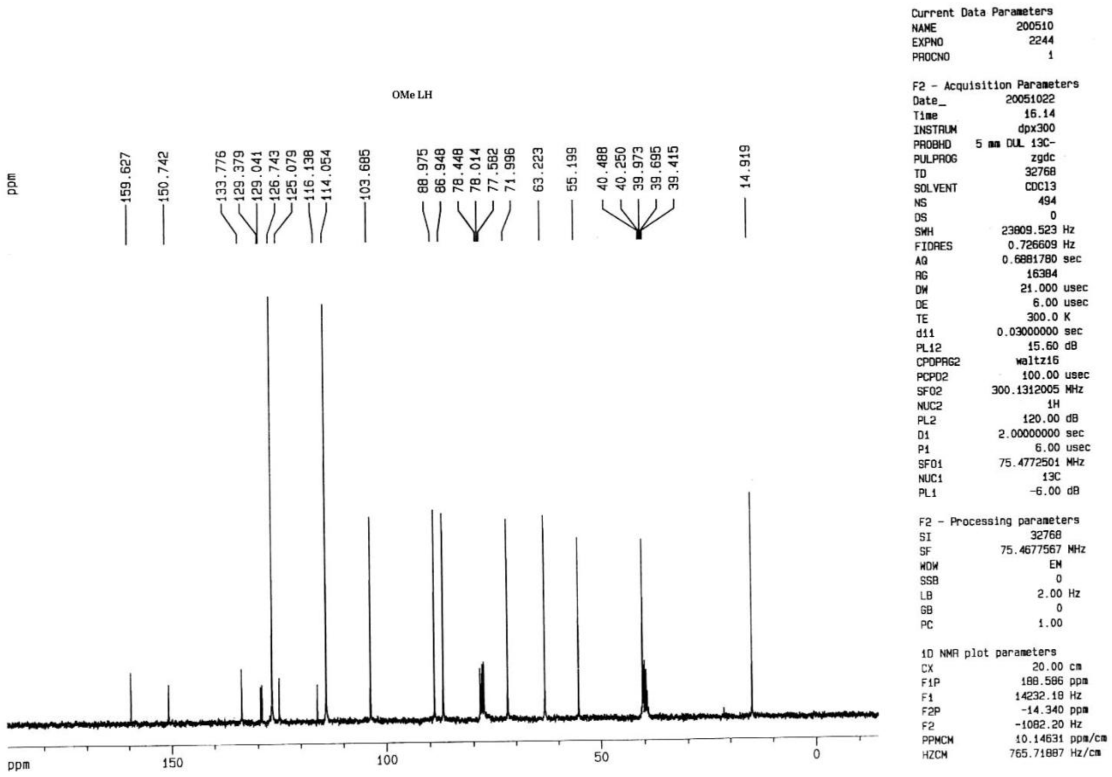

^{13}C NMR spectrum (Bruker, 75.5MHz, mixture of $CDCl_3$ & DMSO) of compound **6b**

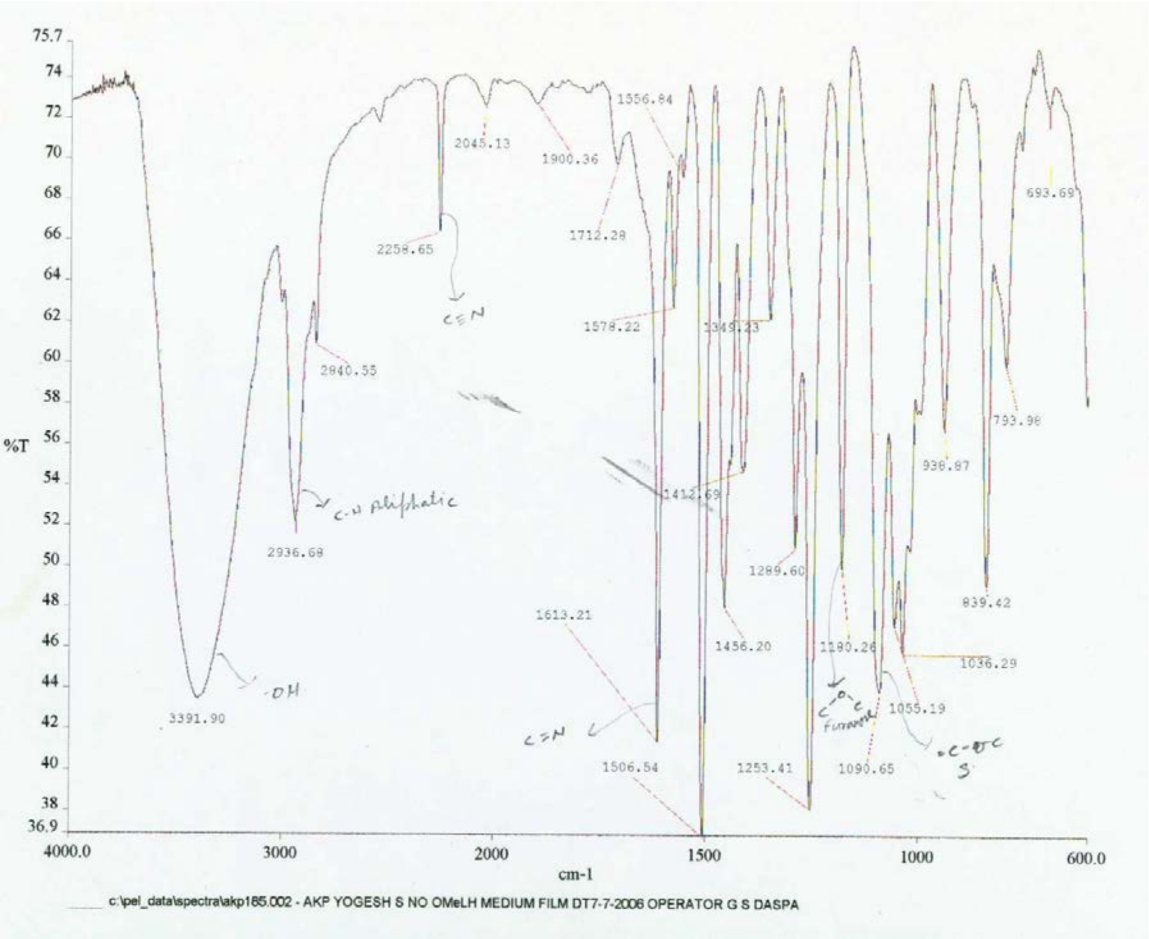

IR spectrum (Perkin-Elmer 2000 FT-IR spectrophotometer) of compound **6b**

2'-Deoxy-1'-(5-cyanomethyl-3-*p*-fluorophenyl)pyrazolyl-*β*-D-ribofuranose (6c)

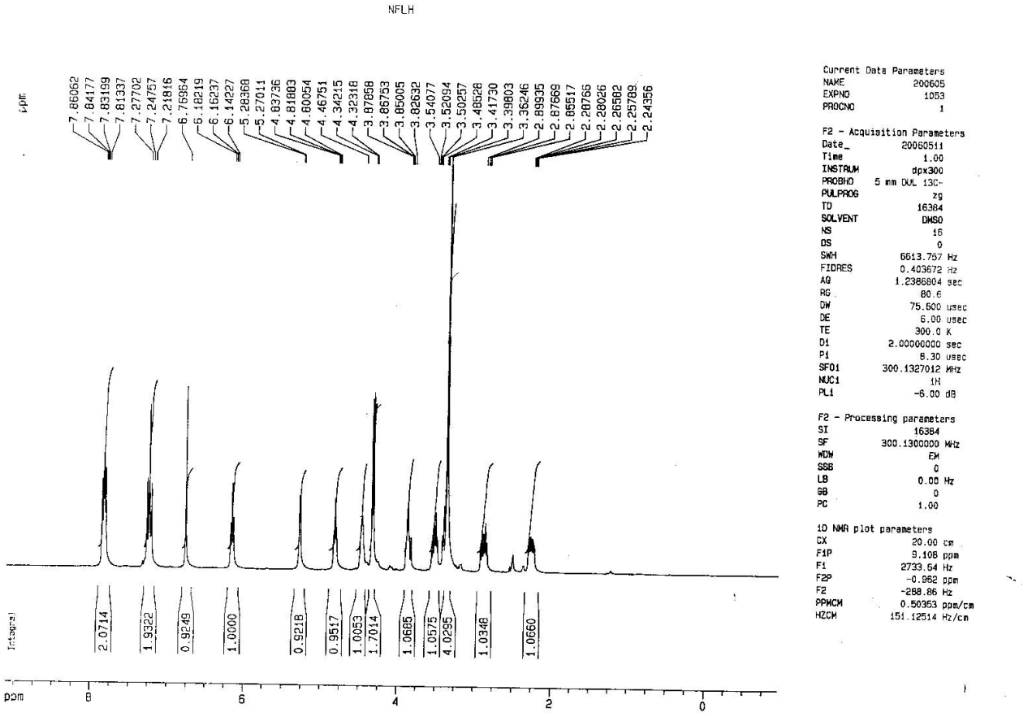

^{1}H NMR spectrum (Bruker, 300MHz, DMSO) of compound **6c**

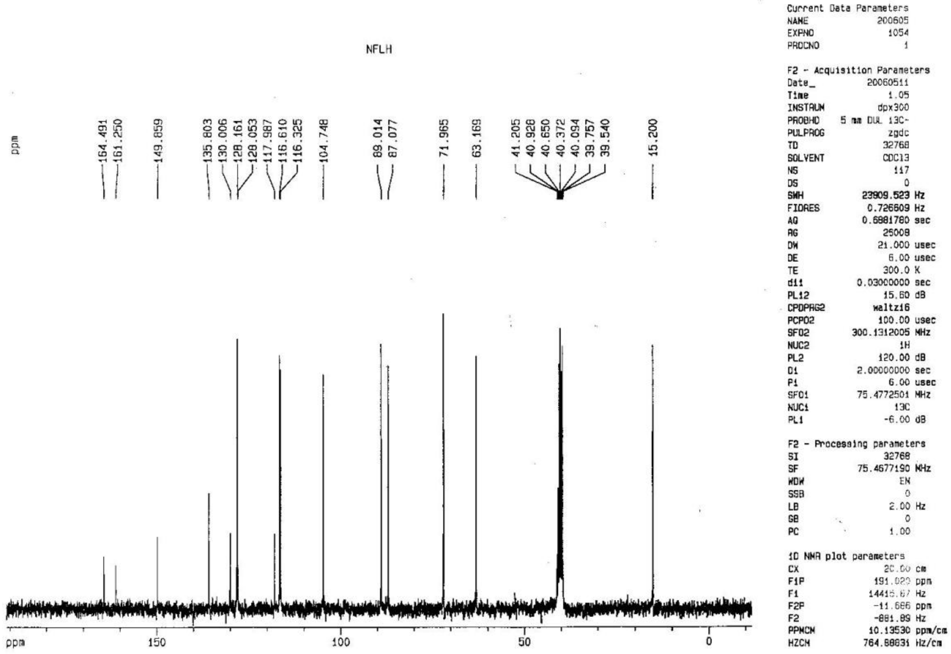

^{13}C NMR spectrum (Bruker, 75.5MHz, $CDCl_3$) of compound **6c**

2'-Deoxy-1'-(5-cyanomethyl-3-*p*-chlorophenyl)pyrazolyl-*β*-D-ribofuranose (6d).

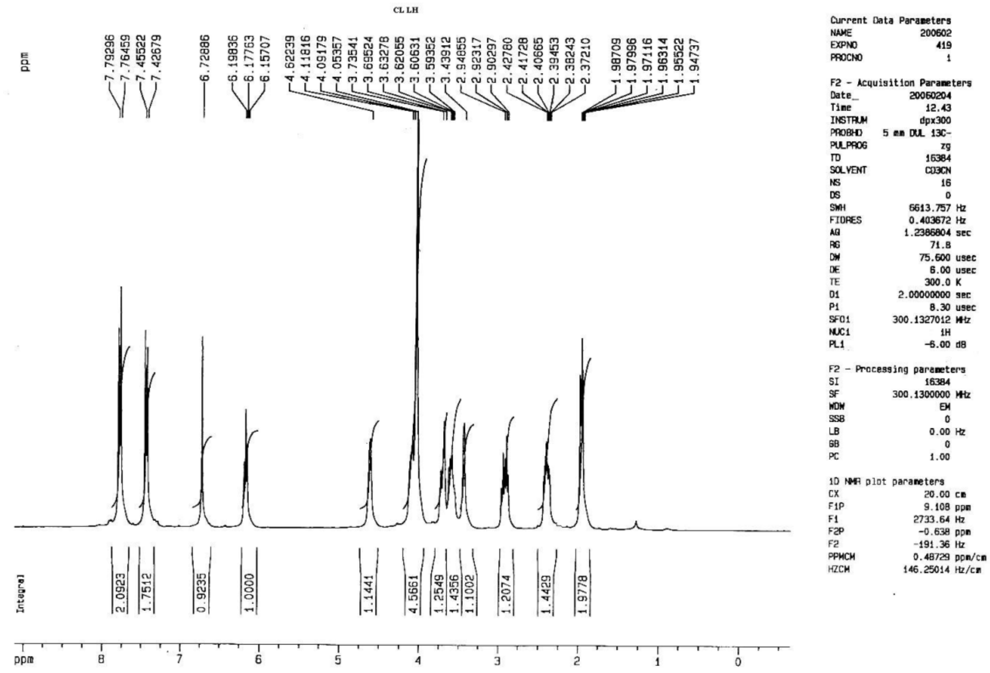

^{1}H NMR spectrum (Bruker, 300MHz, CD_3CN) of compound **6d**

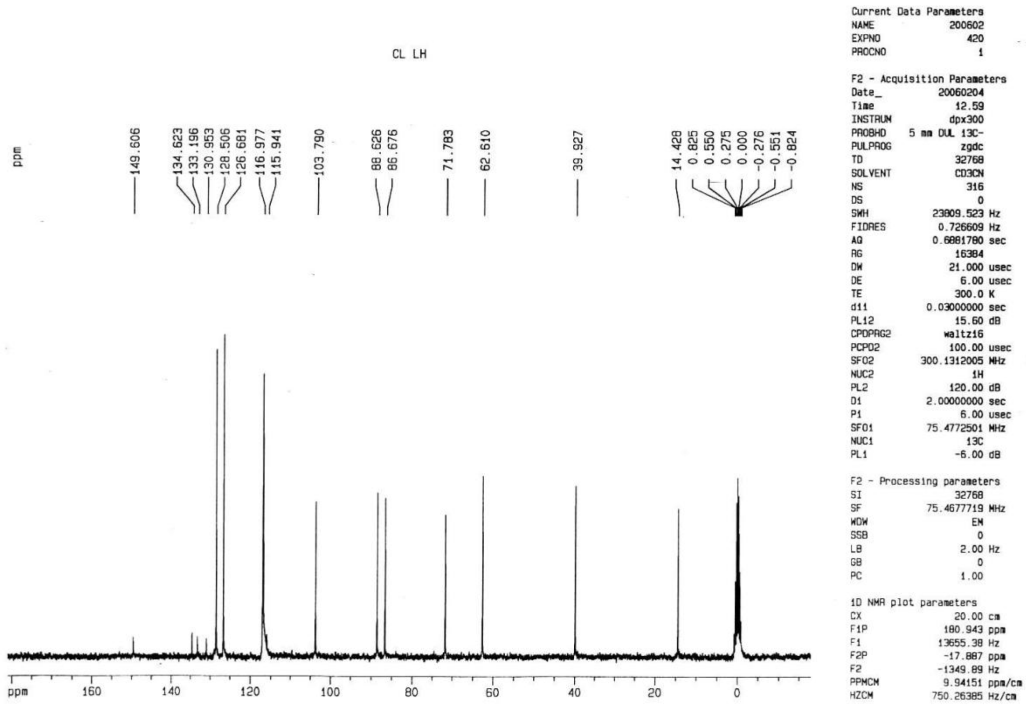

^{13}C NMR spectrum (Bruker, 75.5MHz, CD_3CN) of compound **6d**

2'-Deoxy-1'-(3-*p*-bromophenyl-5-cyanomethyl)pyrazolyl-*β*-D-ribofuranose (6e).

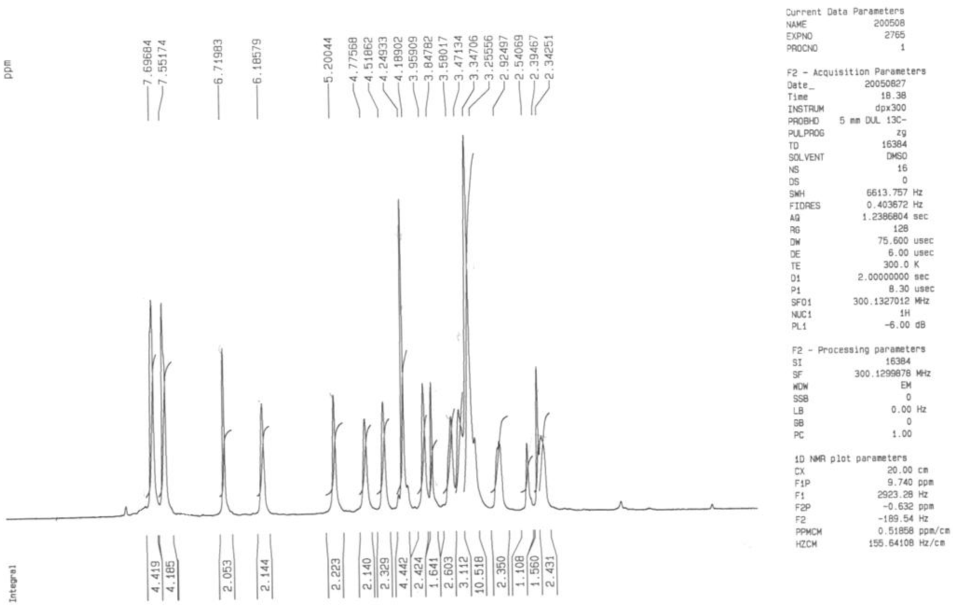

^{1}H NMR spectrum (Bruker, 300MHz, mixture of $CDCl_3$ & DMSO) of compound **6e**

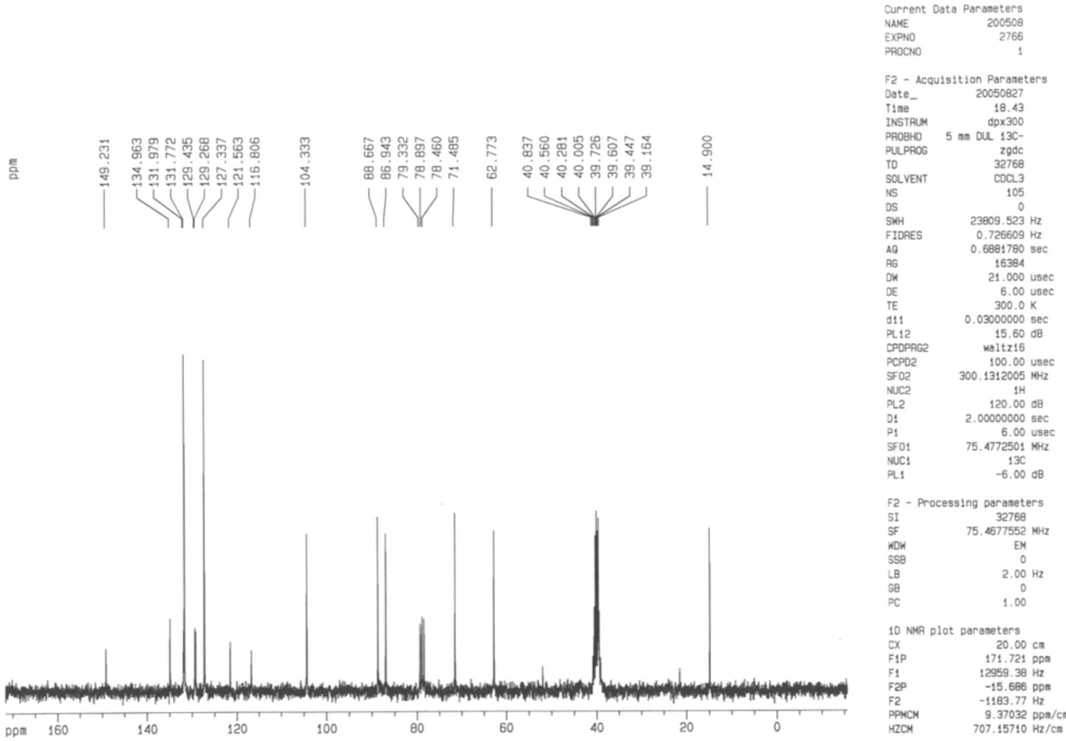

^{13}C NMR spectrum (Bruker, 75.5MHz, mixture of $CDCl_3$ & DMSO) of compound **6e**
